# Keap1-Independent Regulation of Nrf2 Activity by Protein Acetylation and a BET Bromodomain Protein

**DOI:** 10.1371/journal.pgen.1006072

**Published:** 2016-05-27

**Authors:** Nirmalya Chatterjee, Min Tian, Kerstin Spirohn, Michael Boutros, Dirk Bohmann

**Affiliations:** 1 Department of Biomedical Genetics, University of Rochester Medical Center, Rochester, New York, United States of America; 2 Division of Signaling and Functional Genomics, German Cancer Research Center (DKFZ) and Department of Cell and Molecular Biology, Medical Faculty Mannheim, Heidelberg University, Heidelberg, Germany; University of Utah, UNITED STATES

## Abstract

Mammalian BET proteins comprise a family of bromodomain-containing epigenetic regulators with complex functions in chromatin organization and gene regulation. We identified the sole member of the BET protein family in *Drosophila*, Fs(1)h, as an inhibitor of the stress responsive transcription factor CncC, the fly ortholog of Nrf2. Fs(1)h physically interacts with CncC in a manner that requires the function of its bromodomains and the acetylation of CncC. Treatment of cultured *Drosophila* cells or adult flies with *fs(1)h* RNAi or with the BET protein inhibitor JQ1 de-represses CncC transcriptional activity and engages protective gene expression programs. The mechanism by which Fs(1)h inhibits CncC function is distinct from the canonical mechanism that stimulates Nrf2 function by abrogating Keap1-dependent proteasomal degradation. Consistent with the independent modes of CncC regulation by Keap1 and Fs(1)h, combinations of drugs that can specifically target these pathways cause a strong synergistic and specific activation of protective CncC- dependent gene expression and boosts oxidative stress resistance. This synergism might be exploitable for the design of combinatorial therapies to target diseases associated with oxidative stress or inflammation.

## Author Summary

Nrf2-related transcription factors regulate gene expression programs that protect organisms against chemical or oxidative stress. Nrf2-activating drugs hold promise for the treatment of diseases that are connected to oxidative stress or inflammation. We identified Fs(1)h, a bromodomain-containing BET protein, as a negative regulator of Nrf2 function in *Drosophila*. BET proteins are involved in transcription regulation and chromatin organization and have been implicated in several diseases, including cancer. Fs(1)h interacts with acetylated lysines on CncC, the homolog of Nrf2 in *Drosophila*, and thereby prevents target gene activation. Nrf2 can be released from this inhibitory effect by small molecules that specifically interfere with the binding of BET proteins to acetylated targets. Fs(1)h regulates Nrf2 independently of Keap1, a well-studied Nrf2 regulator. Consequently, chemical inhibitors of Keap1 and of Fs(1)h can be combined to achieve synergistic activation of Nrf2 target genes and strongly boost oxidative stress tolerance in *Drosophila*. The Keap1-independent mechanism of Nrf2 regulation is conserved in mammals. We suggest that the synergistic effect of combinatorial Nrf2 targeting drugs may be effective for the treatment of different oxidative stress and inflammation-related diseases.

## Introduction

Nrf2 transcription factors are critically important for the health, homeostasis and longevity of multicellular organisms [1–3]. When cells are confronted with oxidative or chemical stress, Nrf2 stimulates the expression of gene products that protect cell integrity including antioxidants, redox regulators, phase II detoxification enzymes, and factors that maintain proteostasis. The Kelch domain protein Keap1 has been identified as a key mediator of the acute activation of Nrf2 in response to oxidative stress [4, 5]. In unstressed cells Keap1 assembles a Cullin 3-containing ubiquitin ligase complex that targets Nrf2 for proteolysis by associating with its NH_2_-terminally located NEH2 domain. This Keap1-mediated degradation of Nrf2 is relieved upon stress exposure, so that the transcription factor can accumulate in the nucleus and bind to so-called ARE (antioxidant response element) sequences in target gene promoters.

Over the last few years Nrf2 has been implicated in a range biological processes in addition to stress responses. Examples include the regulation of energy metabolism [1, 6], stem cell maintenance [7] and aging [8, 9]. These functions are probably regulated by signals other than toxic insults and presumably require a different transcriptional response, in terms of kinetics and target gene profile. In order to explore how this expanded range of Nrf2 functions might be regulated we conducted a large-scale screen for gene products that are involved in Nrf2 target gene activation.

The experimental model used in our studies is *Drosophila melanogaster*. The fruit fly has an Nrf2 signaling system, which resembles that of mammals [3, 10]. The *Drosophila* ortholog of Nrf2, CncC is encoded by a long splice product of the cap’n’collar gene [8]. The conservation of the Nrf2 pathway genes and its powerful genetic tools make *Drosophila* an excellent model to study this important signaling system.

The *Drosophila* CncC signal transduction pathway can, like its mammalian counterpart, mediate transcriptional responses to various types of chemical or oxidative insults and protect the organism from ensuing damage. CncC can also be activated by dietary dosing with cancer chemo-preventive agents such as oltipraz and sulforaphane [8, 11]. These drugs cause Nrf2 activation without harmful stress and negligible side effects to exposed cells or organisms. Animal experiments have shown these compounds to protect against chemical carcinogens in an Nrf2-dependent manner [12]. Oltipraz and similar drugs exert their effect by interfering with the inhibitory function of Keap1 [13–15].

In a high throughput RNAi screen using *Drosophila* S2 cells [16] we found the gene *fs(1)h* to encode a negative regulator of the *Drosophila* Nrf2 homolog, CncC. Multiple independent double stranded RNAs that target *fs(1)h* mRNA, caused a significant and specific increase in the activity of an ARE luciferase reporter gene [11], identifying the *fs(1)h* gene as a potential inhibitor of Nrf2 function. The product of the *fs(1)h* gene, Fs(1)h, short for female sterile (on the first chromosome) homeotic, is counted as a member of the heterogeneous group of Trithorax proteins which generally function as epigenetic regulators [17, 18]. Fs(1)h is the sole member of the BET protein family in *Drosophila* [19, 20]. BET proteins are characterized by the presence of two bromodomains, adjacent to a so-called extra terminal, or ET, domain [21]. Through the bromodomains, BET proteins specifically bind to polypeptides carrying acetylated lysine residues, including acetyl-histones [22]. Mammalian BET proteins, notably BRD4, have been implicated in the regulation of gene expression. They are known to bind to chromatin and to interact with components of the transcriptional machinery such as P-TEF B and RNA polymerase II [23, 24]. In addition, the activities of specific transcription factors such as NF-κB and Twist can be regulated by direct interaction with BRD4 [25–27]. Similarly, functional experiments and genome-wide ChIP mapping studies suggest that the *Drosophila* BET protein Fs(1)h functions in the regulation of gene activity [18]. Fs(1)h gene products have been found associated with transcription control region and genomic insulator elements [28, 29]. Our experiments show that Fs(1)h can physically interact with CncC to inhibit its transcriptional function. This mechanism is independent of Keap1-mediated Nrf2 regulation.

## Results

### The long isoform of Fs(1)h is a repressor of Nrf2 activity in *Drosophila*

**Fig 1:**
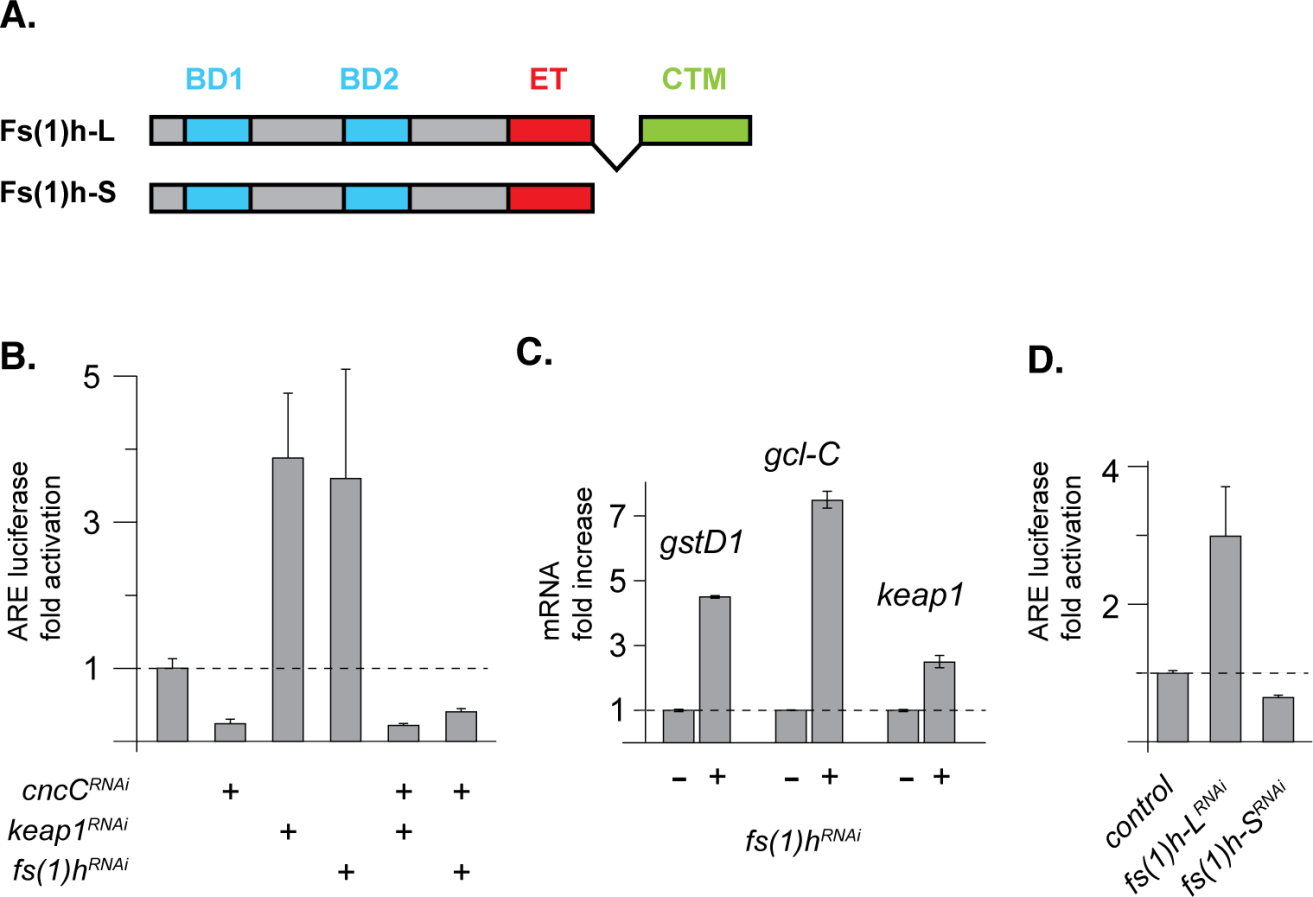
Fs(1)h negatively regulates CncC signaling. (A) The *Drosophila fs(1)h* gene encodes two protein isoforms: the 120kD Fs(1)h-S and the 210kD Fs(1)h-L. Both isoforms contain two bromodomains (BD) and an extraterminal (ET) domain. In addition, Fs(1)h-L carries a unique C-terminal motif (CTM). (B) dsRNA-mediated knock down of Fs(1)h (3.6 fold, P<0.05), like Keap1 knock down (3.9 fold, P<0.01), increases the activity of a transiently transfected ARE-fluc reporter in S2 cells. In both cases, this stimulatory effect is suppressed by CncC knock down. (C) RT-qPCR experiments show that the CncC target genes *gstD1* (P<0.0001), *gclC* (P<0.001) and *keap1* (P<0.05) are activated upon RU486-induced-knock down of Fs(1)h under the control of tub-GS-Gal4 driver. Measurements of transcript abundance levels were normalized to *act5c* transcript levels. Fold activation relative to the mRNA levels in mock treated flies is shown. The error bars indicate standard deviation of 3 biological replicates (flies collected from separate vials). (D) Knock down of Fs(1)h-L alone using a specific dsRNA targeting the CTM region was sufficient to induce ARE-fluc activity in S2 cells (3 fold, P<0.01). However, knock down of Fs(1)h-S alone with dsRNA targeting its 3’ UTR did not induce ARE-fluc activity. Error bars in panels B, C and D signify standard deviation of 3 biological replicates.

A previously described high throughput RNAi screen in *Drosophila* S2 cells had identified Fs(1)h as a possible negative regulator of CncC target gene activity [16]. The function of Fs(1)h as a CncC inhibitor, as suggested by this screen was confirmed by performing transient transfection assays in *Drosophila* S2 cells. These experiments demonstrated that knock down of Fs(1)h caused an increase in ARE reporter gene activity of a similar magnitude as seen upon knock-down of the canonical CncC repressor Keap1 (Fig 1B). When, in addition to Keap1 or Fs(1)h, CncC was knocked down, the activation of the ARE reporter was significantly reduced (Fig 1B). In addition, over-expression of Keap1 to specifically inhibit Nrf2 signaling by limiting its nuclear accumulation, abrogated the induction of the ARE-luciferase reporter in response to Fs(1)h knock down (S5A Fig). Taken together, these indicate that the stimulatory effect of Fs(1)h on ARE reporter is dependent on CncC function. Quantitative mRNA measurements *in vivo* by RT-qPCR supported this conclusion. Flies in which Fs(1)h was knocked down by dsRNA expression under the control of the tub-GS-Gal4 driver showed that endogenous CncC target genes (*gcl-C*, *gstD1*, *keap1*) were up-regulated in *fs(1)h* knock down conditions (Fig 1C).

Like some mammalian BET protein genes, the *Drosophila fs(1)h* locus yields alternatively spliced transcripts that give rise to two different polypeptides, one of 120 and one of 210 kDa molecular mass (Fig 1A) [18, 30]. In the following we will refer to the short and the long isoform as Fs(1)h-S and Fs(1)h-L, respectively. Both alternative splice products contain the two bromodomains and the ET domain. The unique peptide sequences that extend the Fs(1)h-L isoform comprise a C-terminal motif (CTM) (Fig 1A). Genome-wide ChIP experiments have shown that the Fs(1)h-S and L differ substantially in their genomic binding patterns and presumably in their function [20, 28]. To test whether the two splice forms might also differ in their effect on CncC-regulated transcription, we designed dsRNAs that would specifically target only one or the other splice variant (see Materials and Methods). Selective knock down of the long isoform Fs(1)h-L induced ARE reporter activity. However, knock down of the short isoform, Fs(1)h-S failed to do so (Fig 1D). We conclude that the repression of CncC activity is a specific function of Fs(1)h-L. Western blot experiments confirmed the efficient and selective knock down of individual isoforms by the respective dsRNAs (S1A Fig).

**Fig 2:**
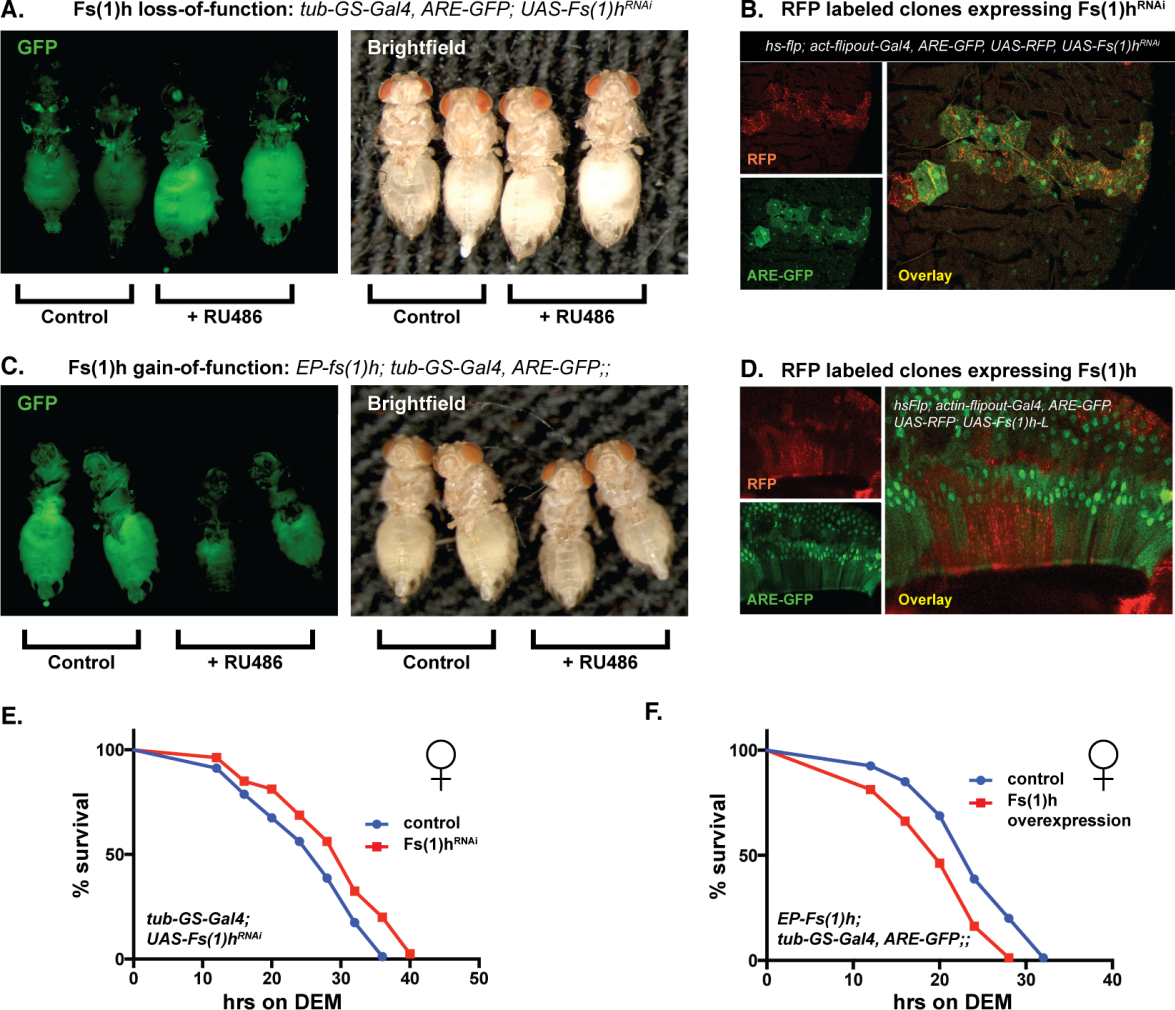
Fs(1)h-L cell-autonomously inhibits CncC activity and affects oxidative stress resistance in adult flies. (A) Ubiquitous knock down of Fs(1)h (in the v51227 RNAi line) in adult flies, using the RU486-inducible tub-GS-Gal4 driver stimulates ARE-GFP reporter activity in most tissues. Two RU486-treated and two mock-treated females are shown in this panel and in panel C. The same flies are shown under UV-illumination to visualize GFP fluorescence and under white light. (B) Knock down of Fs(1)h in actin-flipout-Gal4 clones increased ARE-GFP activity in a cell-autonomous manner in the crop of adult *Drosophila* gut. In the cells in which Fs(1)h expression is knocked down (marked by the expression of RFP, red) ARE-GFP reporter activity (green) is increased. (C) RU486-induced over-expression of Fs(1)h from the EP-fs(1)h allele reduces oltipraz-stimulated ARE-GFP reporter activity in the whole body. Similar effects are seen after ubiquitous over-expression of Fs(1)h-L from a UAS construct (S2 Fig). (D) Fs(1)h-L over-expression in actin-flipout-Gal4 clones (labeled by RFP, red), cell- autonomously reduces ARE-GFP activity in the ejaculatory bulb of adult males. (E) Ubiquitous Fs(1)h knock-down in adult *Drosophila* by inducible expression of a UAS-Fs(1)h^RNAi^ transgene under the control of the tub-GS-Gal4 driver increases oxidative stress resistance. Survival after exposure to 20μM DEM was recorded and the data were analyzed by Mantel-Cox log-rank test. Female flies incubated on RU486 food, showed significantly increased resistance to DEM (P value <0.001) compared to those on control food. A similar effect was observed in males (S3 Fig). The standard deviations of percent survival among biological replicates in this and other stress sensitivity assays are shown in S1 Document. (F) Fs(1)h over-expression increases stress sensitivity. Fs(1)h was over-expressed from the EP-fs(1)h allele in female flies by exposing them to food containing 300μM RU486 for 4 days. Lethality after exposure to 20μM DEM was recorded and analyzed by Mantel-Cox log-rank test. The flies that were kept on RU486 food, showed significantly increased sensitivity to DEM (P value < 0.0001) compared to those on control food. The same experiment was also conducted with males and produced qualitatively the same result (S3 Fig).

The inhibitory function of Fs(1)h on CncC reporter gene activity can also be observed *in vivo*. We conducted experiments with *Drosophila* stocks carrying an ARE-GFP reporter gene in which GFP expression is controlled by four consensus ARE sequences (ARE-GFP, [11]). A UAS *fs(1)h*-RNAi construct was expressed under the control of the RU486-inducible tubulin-GS-Gal4 driver to ubiquitously knock down endogenous *fs(1)h* transcripts in adult flies. This resulted in robust activation of the ARE-GFP reporter transgene in the animals, recapitulating the effect seen in S2 cells. To rule out that the activation of ARE reporter activity was the consequence of an off-target effect, we conducted this experiments with two independent RNAi expression lines and saw similar results (Figs 2A, S1B and S2B).

To examine the *in vivo* effect of *fs(1)h* loss-of-function on the ARE-GFP reporter at the cell level, we conducted knock down experiments in groups of cells using actin-flipout-Gal4, a driver that can be clonally activated by a short heat-shock induced pulse of flp recombinase expression. Clones of RNAi-expressing cells can be distinguished by the expression of an RFP marker. The resulting RFP-labeled *fs(1)h*^*RNAi*^-expressing cells showed increased ARE-GFP reporter activity, consistent with the presumed function of Fs(1)h as a CncC inhibitor. Fig 2B shows such a clone in the crop, a part of the foregut that, together with the anterior midgut, functions as the stomach in *Drosophila* [31]. We found that the epithelial cells of the crop have low basal and high inducible ARE activity. The stimulatory effect of *fs(1)h* knock down was restricted to the cells of the clone, demonstrating that the regulatory function of the protein acts by a cell-autonomous mechanism.

The results of the loss-of-function experiments described above suggested that Fs(1)h can suppress gene activation by CncC. However, an alternative interpretation would be that the observed increase of CncC activity might be an indirect consequence of possible stress or damage in *fs(1)h* loss-of-function conditions. To rule out the latter mechanism, and to demonstrate the repressive function of Fs(1)h directly, we asked whether its over-expression could decrease CncC target gene activity in adult flies. Fs(1)h over-expression was achieved by two alternative strategies. First, we used an EP-line (P(EP)*fs(1)h*[EP439]), a fly stock in which a Gal4 responsive enhancer was integrated in the 5’ genomic region of the *fs(1)h* gene. If combined with the ubiquitously expressed tub-GS-Gal4 driver, expression of endogenous *fs(1)h* mRNA was stimulated when flies were exposed to dietary RU486 (S1B Fig). This resulted in a marked reduction of ARE reporter activity (Fig 2C). Second, we generated a transgenic fly line that expresses a cDNA encoding Fs(1)h-L, the isoform that showed CncC repressor activity in S2 cell-based studies. Inducible expression of the Fs(1)-L under the control of tub-GS-Gal4 caused a clear reduction of ARE-GFP reporter activity (S2A Fig). In addition, over-expression of Fs(1)h-L in RFP-marked hsFlp-induced clones in the ejaculatory bulb of adult *Drosophila*, a tissue with a high basal level of ARE reporter activity, had a negative effect, confirming that Fs(1)h represses CncC in a cell-autonomous fashion (Fig 2D). Thus, Fs(1)h functions as a repressor of CncC’s transcriptional activity both in cell culture and in adult flies.

### Fs(1)h controls oxidative stress defense

The finding that Fs(1)h acts as a repressor of CncC’s transcriptional output, predicted that it should modulate the biological functions of the *Drosophila* Nrf2 ortholog. Nrf2-induced gene expression programs can protect organisms against acute oxidative stress. Experimentally such a stress can be generated by dietary exposure to diethyl maleate (DEM), a glutathione-depleting agent. We have demonstrated previously that genetically increasing CncC activity can boost the resistance of flies against oxidative insults like DEM exposure [8]. To test if Fs(1)h might similarly affect oxidative stress resistance, we measured the DEM sensitivity of flies, in which the activity of Fs(1)h was either suppressed or increased. For loss-of-function experiments, *fs(1)h* mRNA was knocked down in cohorts of young adults flies for 4 days by RU486-induced expression of a corresponding RNAi construct. Subsequently, the animals were transferred to vials containing filter paper laced with a lethal concentration of DEM and the time course of survival was recorded. Cohorts in which *fs(1)h* was depleted displayed a significantly increased survival time as compared to controls (Figs 2E and S3C). For gain-of-function experiments Fs(1)h was over-expressed by feeding EP-fs(1)h; tub-GS-Gal4 stocks with RU486 for 4 days. DEM exposure resulted in a more rapid demise of the flies in these cohorts compared to matching controls (Figs 2F and S3B). Similarly, inducible over-expression in the UAS-Fs(1)h-L fly line also resulted in sensitization to DEM stress (S3A Fig). These DEM exposure experiments support the conclusion that Fs(1)h is a significant regulator of stress defense mechanisms, presumably through its effect on CncC.

### Mechanism of CncC regulation by Fs(1)h

**Fig 3:**
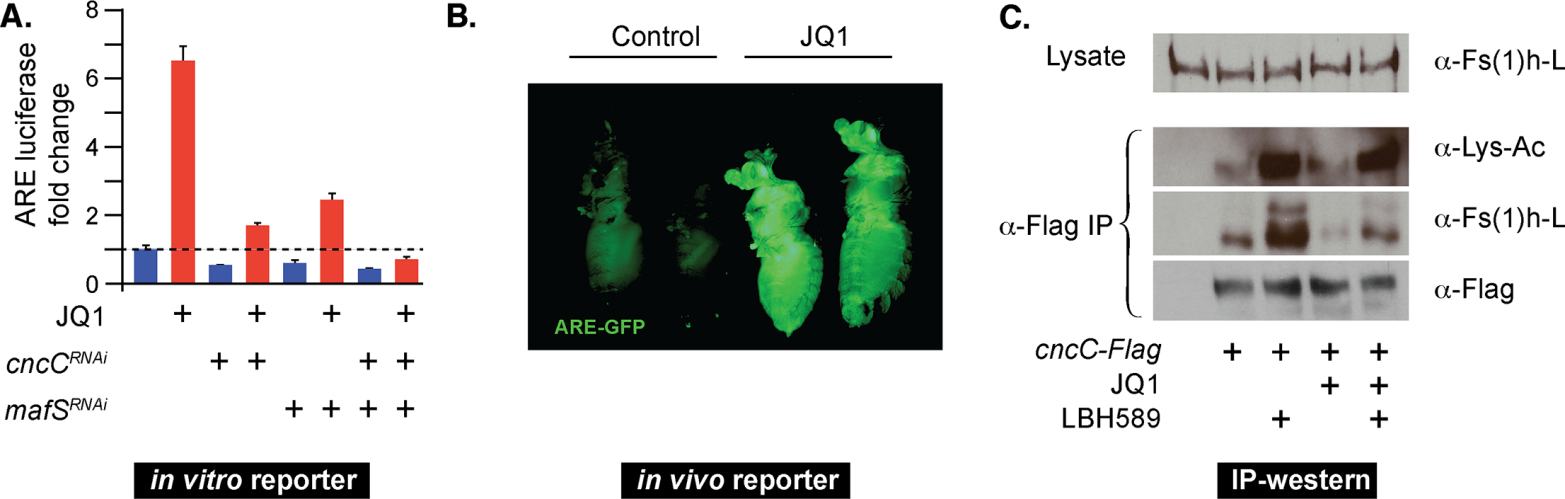
Bromodomains mediate the inhibition of CncC by Fs(1)h. (A) S2 cells transfected with the ARE-fluc reporter plasmid were treated with 1μM JQ1 for 24 hrs, which stimulated ARE-fluc activity (6.5 fold, P<0.001). This increase was strongly diminished after knock down of CncC (JQ1 mediated induction is 3.2 fold, P<0.001) or MafS (JQ1 mediated induction is 4.2 fold, P<0.002). Combined knock down of MafS and CncC almost completely eliminates the JQ1 effect (JQ1 mediated induction is 1.6 fold, P>0.05). The diagram shows the fold change in luciferase activity relative to controls. Error bars indicate standard deviation of 3 biological replicates. (B) 5 day old adult ARE-GFP flies were maintained on food containing 0.25 mM JQ1 for 2 days. Fluorescence images of adult flies showed a strong induction of ARE-GFP reporter activity by JQ1. Two randomly chosen JQ1-treated and two solvent-treated female flies are shown. (C) Co-immuno-precipitation of endogenous Fs(1)h-L with over-expressed CncC-Flag in S2 cells. S2 cells were transfected with either actin-Gal4 plasmid alone (lane 1) or with actin-Gal4 and UAS-CncC-Flag plasmids (lanes 2–5). The cells over-expressing CncC-Flag were treated either with the HDAC inhibitor LBH589 (500nM) and/or JQ1 (10μM) as indicated, or with 0.01% DMSO (vehicle) for 6 hours before they were processed for immuno-precipitation. 10μM JQ1 was also added to the lysate from cells treated with JQ1 to assess the effect of JQ1 on Fs(1)h-CncC interaction. Immuno-precipitation was performed using anti-Flag antibody followed by immuno-blotting with anti-Fs(1)h-L antibody. The acetylation status of CncC in the same immuno-precipitates was examined in western blots using an antibody against acetylated lysine.

Through their double bromodomains BET proteins like Fs(1)h can bind to polypeptides that carry acetylated lysine residues. To assess whether acetyl lysine binding might contribute to the repressive function of the protein towards CncC’s transcriptional activity, we conducted experiments with JQ1, a specific inhibitor of BET-domain interactions with acetylated substrates [32]. Treatment of S2 cells with JQ1 caused a strong activation of ARE-luciferase reporter activity that was markedly reduced by knock down of either CncC or its obligate heterodimerization partner MafS (Fig 3A). Consistent with the observation that dsRNA-mediated knock down of either CncC or MafS, individually does not completely eliminate the targeted transcripts, a residual response of the reporter to JQ1 could be observed. To confirm the conclusion that the active form of *Drosophila* Nrf2, the MafS-CncC heterodimer, specifically mediates the effect of JQ1 we combined CncC and MafS knock down conditions. Under these conditions, the JQ1-mediated induction of the ARE reporter was almost completely abrogated. This observation confirms the specificity of the JQ1-effect and further supports our conclusion that Fs(1)h affects ARE activity by interfering with CncC function. Similarly, dietary JQ1 exposure (Fig 3B) resulted in a strong enhancement of ARE reporter activity also in flies. This stimulatory effect does not seem to be a stress response, as we did not detect any increased mortality in flies or cells after treatment with JQ1 as they would result from treatment with Nrf2-inducing stressors such as paraquat or DEM.

The experiment described above suggests that JQ1 can relieve the inhibition of CncC by Fs(1)h. Since JQ1 has been shown to block the interaction of BET bromodomains with acetyl lysine residues, we proposed that Fs(1)h would interact with acetylated CncC and that this interaction would be disrupted by JQ1, causing an increase in CncC activity. Indeed, specific acetylated forms of mammalian Nrf2 have been described [33, 34]. To assess whether CncC might also be a substrate for acetylation and could therefore represent a potential binding partner for the Fs(1)h bromodomains, we performed immune precipitation/western blot experiments. S2 cells expressing a Flag-epitope tagged version of CncC were processed for immuno-precipitation with an anti-Flag antibody. Staining western blots of the resulting immuno-precipitates with a generic anti-acetyl lysine antibody revealed that, like its mammalian counterpart, CncC protein is acetylated (Fig 3C). Treatment of the cells with the broad-spectrum HDAC inhibitor LBH589 increases the acetylation of the immuno-precipitated CncC, confirming that the protein is a substrate for acetylation / deacetylation reactions *in vivo*.

Next, we tested whether Fs(1)h might physically interact with CncC and, if so, whether Fs(1)h binding correlates with CncC acetylation status. Confirming this idea, we found that Flag-tagged CncC expressed in Schneider cells could be co-immuno-precipitated with endogenous Fs(1)h-L. The yield of the recovered Fs(1)h-L increased in the presence of HDAC inhibitor and decreased in the presence of JQ1 (Fig 3C). These results indicated that Fs(1)h-L and CncC bind to each other through a BET bromodomain–acetyl lysine interaction.

**Fig 4:**
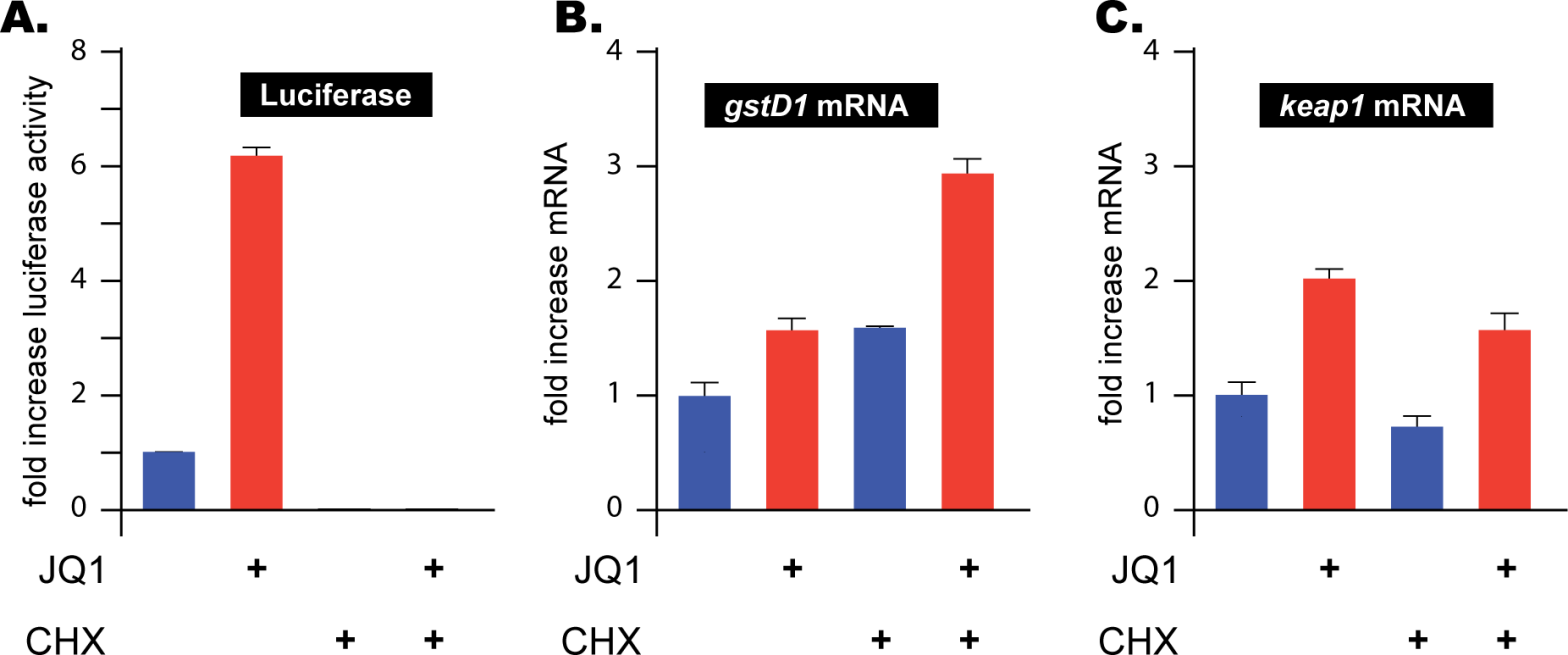
Stimulation of CncC target gene expression is independent of *de novo* protein synthesis. (A) S2 cells, transfected with the ARE fluc reporter were pre-treated with cycloheximide (CHX) or mock-treated for 4 hours and then exposed to 1 μM JQ1 or DMSO as a solvent control for 8 hrs. as indicated in the figure. In the absence of CHX luciferase activity was easily detectable and was strongly responsive to JQ1 treatment. No luciferase activity was detectable in CHX-treated cells, both in basal and JQ1-treated conditions. The absence of luciferase indicates that inhibition of *de novo* protein synthesis in this experiment is efficient. The averages of ARE-luciferase activity in biological replicates are shown here. (B and C) The expression of two CncC-regulated mRNAs (*gstD1* and *keap1*) remained JQ1 inducible in in the absence (P<0.05 and P<0.01 respectively for *gstD1* and *keap1*) and the presence of CHX (P<0.01 and P<0.02 respectively for *gstD1* and *keap1*). Note that *gstD1* expression, like that of many other stress-inducible genes [35, 36] increases in the presence of CHX. Nevertheless, the JQ1 response remains robust under CHX treatment also in this case. The mRNA levels were normalized to *actin5C* transcript levels. The error bars indicate standard deviation of 3 biological replicates.

Next we wanted to investigate whether the stimulatory effect of Fs(1)h inhibition on CncC target gene expression might be mediated by changes in the expression of CncC or some other gene. Therefore, we treated S2 cells with the protein synthesis blocker cycloheximide prior to JQ1 exposure. Under those conditions, protein synthesis, as measured by luciferase expression (Fig 4A), was completely inhibited, but the JQ1-mediated induction of the CncC target genes *gstD1* and *keap1* was unaffected (Fig 4B and 4C). We conclude that Fs(1)h regulates CncC at the post-translational level to affect the expression of its target genes. Such a model fits our hypothesis that Fs(1)h regulates CncC protein function by physically interacting with it.

The *cnc* gene generates several different protein products by alternative splicing [37, 38]. The three initially described Cnc splice forms CncA, CncB and CncC all share a common C-terminal region that comprises the bZIP dimerization and DNA-binding domain. Moving from the A to the C isoform the polypeptides include progressively more N-terminal sequences. Of these three gene products only CncC contains the NEH2 domain, which mediates binding to and repression by Keap1 [8]. To assess which sequences are required for binding to Fs(1)h we performed co-immuno-precipitation experiments with different Cnc protein isoforms. Epitope-tagged versions of CncA, B, and C were expressed in S2 cells and immuno-precipitated with an anti-Flag antibody. The immuno-precipitates were analyzed by western blot probed with an anti Fs(1)h antibody that recognizes both the short and the long isoforms of Fs(1)h. Endogenous Fs(1)h-L protein was only detected in the immuno-precipitated material from S2 cells that expressed the longest isoform, CncC. However, it was not co-precipitated with CncA or CncB (S4 Fig). Notably this behavior is shared by Keap1, which also binds to CncC only but not to the two shorter isoforms. No interaction between CncC and Fs(1)h-S was identified in this experiment. This is consistent with our finding that only Fs(1)h-L is involved in the negative regulation of CncC. We conclude that sequences within the region of amino acids 1–578 which are unique to CncC, the only isoform with recognized functions in stress response, are required for both Fs(1)h-L and Keap1 binding. Further experiments are required to test if the binding of the two inhibitory proteins, Keap1 and Fs(1)h can happen simultaneously or is mutually exclusive [25–27]. Interestingly, we did detect the short isoform Fs(1)h-S as in the co-precipitate with CncB (S4 Fig). CncB is not involved in stress responses, but functions in embryonic patterning and development. It has been suggested to act as a negative regulator of transcription [10, 37]. The functional relevance of a potential interaction between CncB and Fs(1)h-S awaits experimental analysis.

### Fs(1)h is a part of a conserved, Keap1-independent axis of Nrf2 regulation

**Fig 5:**
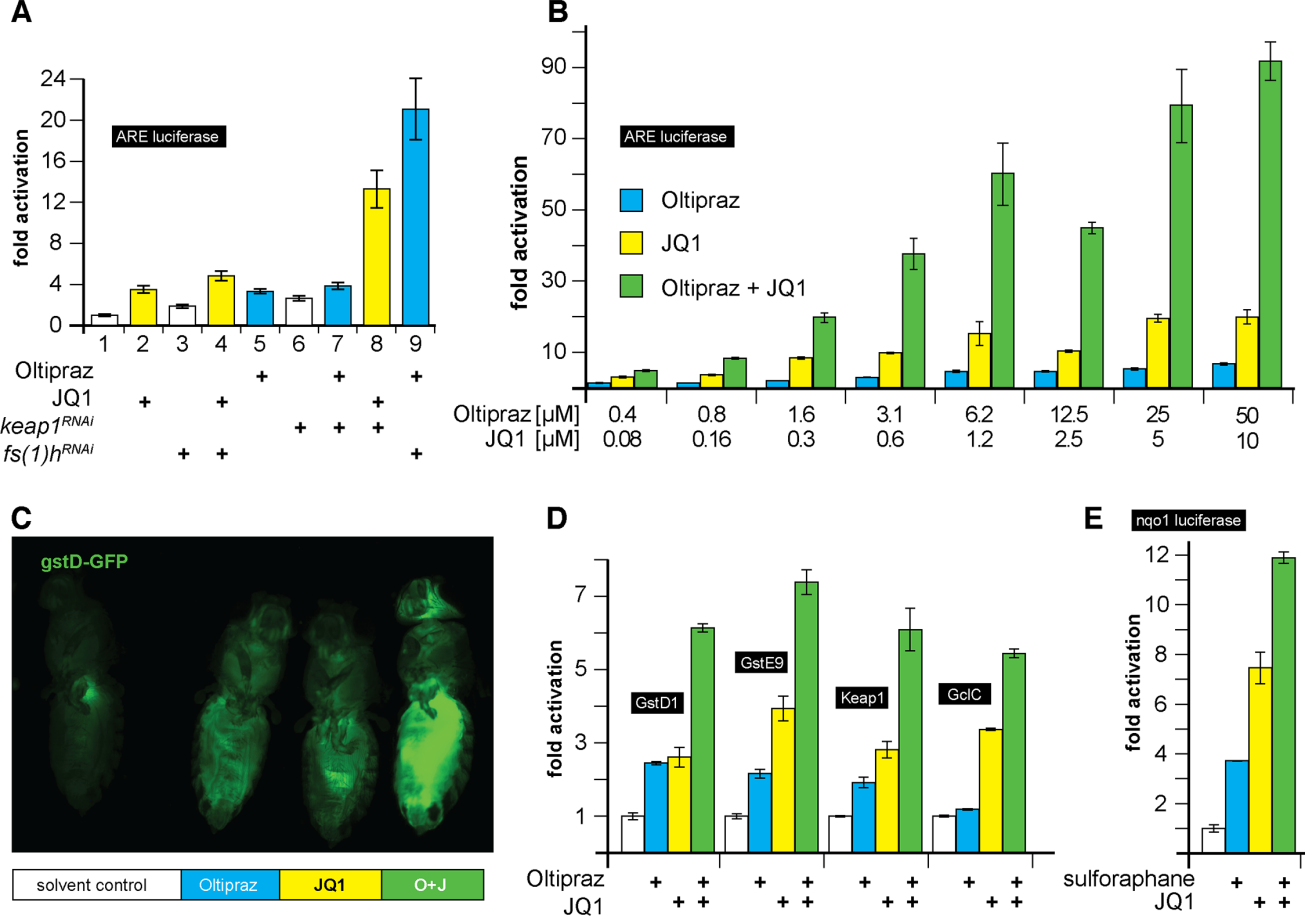
Fs(1)h regulates CncC signaling independently of Keap1. (A) The graph shows the effect of different combinations of Keap1 RNAi and Fs(1)h RNAi with JQ1 or oltipraz, on ARE-fluc activity in S2 cells. Treatment of cells with Keap1 RNAi or oltipraz relieves the same mechanism of CncC inhibition and combining both manipulations does not have a stronger effect than either treatment by itself. The same applies to Fs(1)h knock down and JQ1. However, combining Fs(1)h inhibition by RNAi or drug treatment with Keap1 inhibition causes a strong cooperative activation of ARE-Fluc. Fluc measurements were normalized to actin renilla-luciferase expression, which was used as an internal control. (B) Combination of different concentrations of oltipraz and JQ1 leads to synergistic activation of ARE-Fluc reporter in S2 cells. (C) 5 day old gstD-GFP flies were exposed to food containing 0.4 mM oltipraz and/or 0.1mM JQ1 for 4 days. Fluorescence images of adult flies showed a stronger induction of the gstD-GFP reporter by combined treatment with oltipraz and JQ1. One randomly chosen female fly from each group is shown. (D) Combined effect of oltipraz and JQ1 on CncC target gene expression. S2 cells were treated with 25μM oltipraz and/or 1μM JQ1 as indicated. *gstD1*, *gstE9*, *keap1* and *gclC* mRNAs were quantitated by RT-qPCR. Graphs show fold induction of mRNA levels of CncC target gene after normalization to *actin5C* expression. The error bars indicate standard deviation of 3 biological replicates. (E) Cooperation of the Keap1 inhibitor sulforaphane and BET protein inhibitor JQ1 in mammalian cells. HEK293 cells that were transiently transfected with the Nrf2-responisve NQO1-fluc reporter were treated separately with 10μM sulforaphane (a Keap1 inhibitor similar to oltipraz that is suitable for mammalian cell culture), with 0.5μM JQ1 or with a combination of both. Combined treatment with sulforaphane and JQ1 showed more than additive induction of NQO1-fluc compared to individual treatments. The firefly luciferase activity was normalized to renilla-luciferase activity expressed under the control of a thymidine kinase promoter. Error bars in panels A, C, and D represent standard deviation of 3 biological replicates.

The discovery that Fs(1)h physically interacts with and inhibits CncC raised the question of how this activity relates to the regulatory function of Keap1, the canonical inhibitor of Nrf2 proteins. Specifically, we wanted to determine whether Fs(1)h acts via Keap1-mediated CncC degradation, or through an independent mechanism to control target gene expression. To address this question we conducted transfection experiments in S2 cells with drugs and RNAi’s that specifically interfere with Keap1 or Fs(1)h functions (Fig 5A). The experiments described above indicated that JQ1 prevents the inhibitory effect of Fs(1)h on CncC, thereby stimulating ARE-dependent gene expression. Consistent with this interpretation, *fs(1)h* knock down and JQ1 exposure stimulate ARE-luciferase reporter activity to a similar degree (Fig 5A, compare lane 1 with 2 and 3), and combining the *fs(1)h* RNAi with JQ1 treatment does not further enhance this activity (Fig 5A, lane 4). Likewise, oltipraz exposure and Keap1 knock down activated the reporter to comparable levels, yet the effect of the two treatments is non-additive, confirming that oltipraz acts specifically through inhibition of the Keap1-CncC interaction (lanes 5–7). Interestingly, however, eliminating the function of both Fs(1)h and Keap1 simultaneously by combined RNAi and drug treatments caused a synergistic activation of the reporter (lanes 8 and 9). Similarly, combining JQ1 and Oltipraz treatment resulted in a synergistic activation of the ARE reporter in S2 cells over a range of concentrations (Fig 5B). Confirming the results from S2 cells, the combined treatment of adult flies with oltipraz and JQ1 lead to stronger induction of the gstD-GFP reporter relative to the effects of either oltipraz or JQ1 alone (Fig 5C). *gstD1* is a target gene of CncC and a gstD-GFP reporter had previously been generated by placing a GFP transgene under the control of a *gstD1* promoter fragment [8].

The induction of gstD-GFP reporter activity in flies in response to JQ1 treatment also indicated that natural CncC regulated enhancers, and not only synthetic ARE reporters are responsive to Fs(1)h inhibition in adult *Drosophila*. Mirroring the measurements of luciferase reporter constructs, the expression of four conserved Nrf2 target genes *(gstD1*, *gstE9*, *keap1*, *gclc)* was inducible by treatment with JQ1 or oltipraz and combined treatment had a more than additive effect relative to the response to the individual drugs (Fig 5D).

In further experiments we found that fs(1)h knock down also led to a synergistic activation of ARE luciferase reporter activity when combined with either Keap1 knock down or CncC over-expression. Presumably, CncC over expression overwhelms the capacity of Keap1 to target the protein for degradation. Thus, both in conditions of CncC over-expression and Keap1 knock down, CncC can accumulate in the nucleus. Gene activation by this increased nuclear pool of CncC protein would then be under negative control by Fs(1)h (S5A Fig). Taken together, these results indicate that Fs(1)h and Keap1 regulate Nrf2 function through independent mechanisms.

The effective and specific pharmacological activation of the Nrf2 response by combined JQ1 and oltipraz treatment might be therapeutically beneficial, provided that the mechanisms that we have characterized in *Drosophila* are conserved and operational in mammals. Interestingly, it has been shown in recent publications [39, 40] that mammalian Nrf2 can be stimulated by JQ1. We therefore tested if mammalian cells displayed cooperative activation of Nrf2 target genes in response to the combinatorial treatment that we had found effective in *Drosophila*. Confirming this idea, combined treatment of human HEK293 cells with sulforaphane, a Keap1 inhibitor functionally similar to oltipraz, and JQ1 results in a more than additive activation when compared to individual treatments (Fig 5E). In addition, JQ1 but not sulforaphane caused synergistic activation of NQO1-luciferase reporter when combined with over-expressed Nrf2, indicating that the Keap1-independent regulation of Nrf2 by BET protein is conserved in mammals (S5B Fig). Combined dosing of these two drugs might therefore open new avenues for treatment of diseases where increased Nrf2 activity could be beneficial.

### Combinatorial effect of oltipraz and JQ1 on oxidative stress tolerance

**Fig 6:**
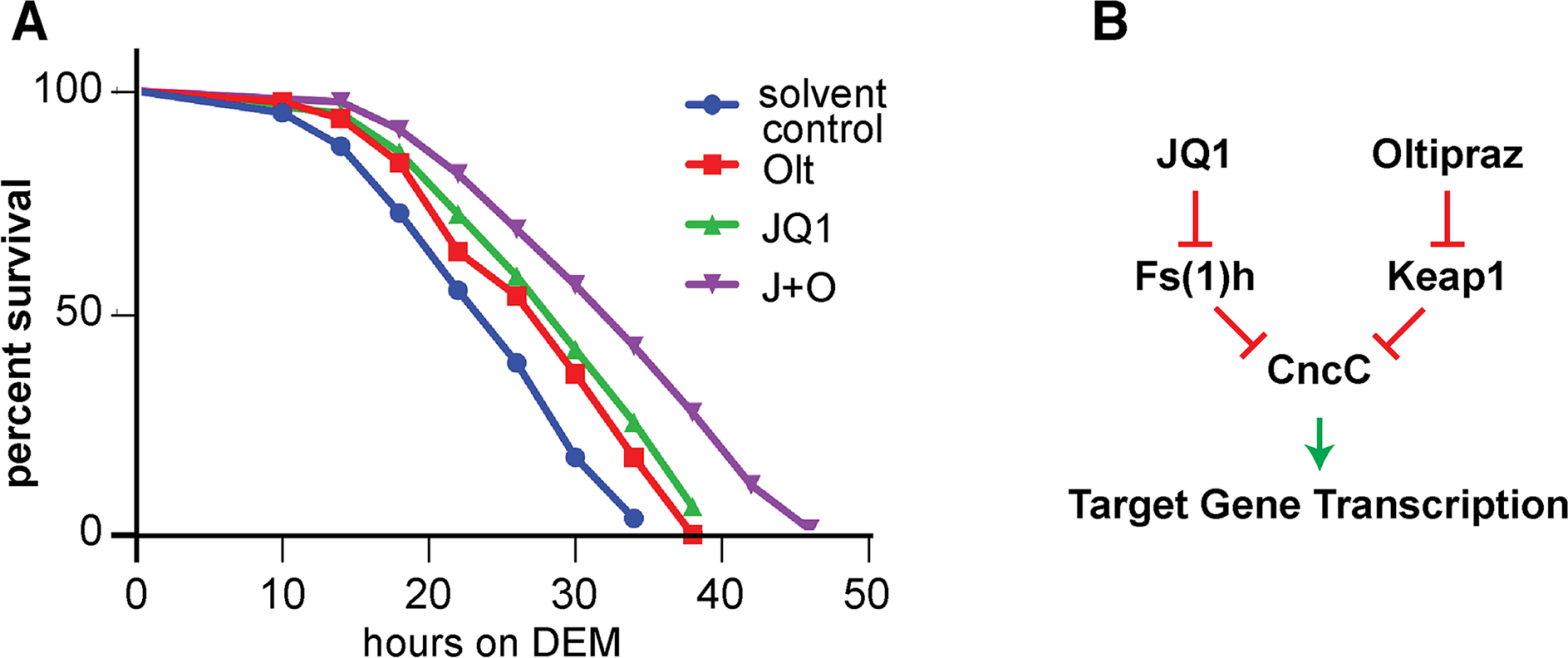
Simultaneous inhibition of Keap1 and BET proteins impart enhanced oxidative stress tolerance. (A) 5 day old female *w*^*1118*^ flies were placed on food containing 0.4mM oltipraz and/or 0.1mM JQ1 for 4 days and then were exposed to 20μM DEM. Survivorship was assessed. Mantel-Cox log-rank test showed that pre-treatment with either oltipraz or JQ1 significantly increased oxidative stress tolerance (P value <0.005 for control/oltipraz comparison and P value <0.001 for control/JQ1 comparison). It was also found that pre-treatment with both oltipraz and JQ1 extended survival after DEM exposure significantly more than pre-treatment with either drug alone (P value <0.001 for olt/combined comparison and P value <0.005 for JQ1/combined comparison). Qualitatively identical results were found when males were used (S3D Fig). (B) Proposed model for synergistic activation of Nrf2 signaling by oltipraz and JQ1. CncC is regulated independently by Keap1 and Fs(1)h. Treatment of cells with the Keap1 inhibitor oltipraz relieves a different mechanism of CncC inhibition from that relieved by the BET protein inhibitor JQ1. Combining both manipulations, therefore, has a stronger effect than either treatment by itself and causes a cooperative activation of CncC target genes.

If a combinatorial treatment with the Keap1 inhibitor oltipraz, and the BET bromodomain inhibitor JQ1 activates Nrf2-dependent stress defense and antioxidant gene expression programs more strongly than oltipraz or JQ1 alone, we would expect a further increase in the resistance of flies to acute oxidative challenges. To confirm this prediction we exposed adults to a lethal dose of DEM after they had been kept on food containing oltipraz, JQ1 or both drugs for 4 days. Monitoring the time course of mortality showed that pretreatment with oltipraz and JQ1 alone enhances the oxidative stress tolerance when compared to the control group. However, pretreatment with a combination of oltipraz and JQ1 increased survival times even further (Figs 6A and S3D). Thus, combinatorial treatment with oltipraz and JQ1 can yield effective protective effects against oxidative challenges and may also be useful for therapeutic applications in which Nrf2 function is expected to be beneficial.

## Discussion

BET proteins including *Drosophila* Fs(1)h and the members of the mammalian Brd family function as epigenetic readers and transcriptional regulators [28, 29, 41, 42]. They can affect the function of specific transcription factors such as NF-kB, FosL and Twist, but also interact with components of the general transcription machinery such as P-TEFb [23–27, 43]. Mammalian BET proteins, most notably Brd4, have raised broad biomedical interest as well, because they can support several types of cancer, including NUT midline carcinoma, multiple myeloma and acute leukemia [44–47]. The implication of BET proteins in these malignancies could have clinical impact as the availability of specific inhibitors makes them potentially promising drug targets. In order to evaluate and exploit these opportunities, it is critical to gain a better understanding of the complex functions and molecular targets of BET proteins.

We have identified the *Drosophila* BET protein Fs(1)h as a novel inhibitor of the *Drosophila* Nrf2 homolog CncC. The *fs(1)h* gene encodes two different isoforms, Fs(1)h-S and Fs(1)h-L. Fs(1)h-S has previously been reported to transcriptionally activate the *ultrabithorax (ubx)* gene, a function that is not shared by the larger Fs(1)h-L isoform [18]. Conversely, our study showed that only Fs(1)h-L, but not Fs(1)h-S, has the potential to inhibit CncC target gene activation. The divergence of function between Fs(1)h-S and L is also reflected in their respective genomic binding patterns: A recent genome-wide ChIP-seq study showed that Fs(1)h-S and Fs(1)h-L are present at different locations throughout the *Drosophila* genome [28]. Whereas Fs(1)h-S was enriched in promoters and enhancers, Fs(1)h-L was predominantly localized to the insulators. Interestingly, our analysis of the same ChIP-seq data revealed that Fs(1)h-L also localizes to the promoter region of many CncC target genes such as *gstD1*, *gclC*, and *keap1*. It is possible that Fs(1)h-L interacts with promoter-bound CncC to prevent the induction of its target genes.

Nrf2 is an attractive drug target. Compounds such as oltipraz and sulforaphane which interfere with the Keap1-Nrf2 interaction to activate target genes without causing cell stress, are effective as cancer chemo-preventive agents in animal experiments [48–51]. Nrf2-activating drugs can also be beneficial in the treatment of other diseases that are associated with oxidative stress, including neurodegenerative and inflammatory conditions [52, 53]. Indeed, an Nrf2 inducer, dimethyl fumarate, marketed under the brand name Tecfidera, has recently gained FDA approval for treatment of multiple sclerosis [54, 55]. The identification of JQ1 as a CncC activator and, the discovery that JQ1 and oltipraz synergistically activate Nrf2 dependent gene expression programs in *Drosophila* and mammalian cells suggest innovative therapeutic options. Conventional Nrf2 activating drugs like sulforaphane and oltipraz, though effective in some cases, show moderate induction of Nrf2 and often have off-target effects [49, 52]. Combinations of JQ1 and oltipraz-like drugs might stimulate Nrf2 dependent protective gene expression more efficiently than either compound alone, or could achieve beneficial effects at lower doses, thereby decreasing the risk of unwanted side effects.

Based on the known properties of Fs(1)h and Keap1 and our cell culture and biochemical experiments we suggest a mechanisms by which Fs(1)h represses CncC function. As opposed to the effect of Keap1, which targets CncC for cullin 3-mediated proteasomal degradation in the cytoplasm, the inhibitory, acetylation-dependent interaction between Fs(1)h and CncC presumably occurs in the nucleus where the BET protein resides (Fig 6B). The independent mechanisms of CncC inhibition by Keap1 and Fs(1)h are consistent with the synergistic effect of combined JQ1 and oltipraz treatment on Nrf2 target gene expression.

*fs(1)h* is the only BET protein encoding gene in *Drosophila* and Brd4 is the closest ortholog of Fs(1)h in mammals [28]. Recent publications by Michaeloudes et al and Hussong et al have implicated Brd4 in the regulation of mammalian Nrf2 [39, 40]. In agreement with our conclusions, Michaeloudes and colleagues have found that Brd4 and other mammalian BET proteins namely Brd2 and Brd3, physically interact with Nrf2. Hussong and colleagues, on the other hand, suggested a somewhat different model of Nrf2 regulation by Brd4. They reported that JQ1-treatment of human cells activates Nrf2 target genes, but suppresses Keap1 expression. Accordingly, they reason that the activation of Nrf2 target genes by JQ1 might be mediated by a loss of Keap1 repression [40]. The data we generated in *Drosophila* S2 cells, on the other hand, show that both Fs(1)h knock down and JQ1 treatment leads to increased *keap1* transcription, which is consistent with the previous identification of the Keap1 gene as a target of CncC mediated feedback regulation. It is possible that species- or cell type-specific differences in JQ1-Keap1 cross talk exist and account for these divergent results. In any case, our finding that JQ1 and sulforaphane synergistically activate an ARE reporter in human cells supports the notion that combinatorial treatment with these two drug types might be therapeutically beneficial.

Our data show that the suppression of CncC activity by Fs(1)h relies on a bromodomain–acetyl lysine interaction. This finding implies a repressive function of acetylation on CncC’s transactivation potential. Such a conclusion appears to be at odds with reports showing acetylation of Nrf2 by CBP to increase its binding to target DNA and to enhance target gene transcription [33, 34]. However, Mercado et al. have reported that inhibition of HDAC2 and the resulting increase in Nrf2 acetylation can suppress Nrf2-mediated target gene induction and antioxidant defense in chronic obstructive pulmonary disease (COPD) [56]. It seems therefore that Nrf2 acetylation can have positive as well as negative effects on the transcriptional function of Nrf2 proteins. Further studies are needed to uncover whether specific acetylation marks on Nrf2 lead to either activation or suppression of its transcriptional activity and whether BET proteins can selectively interact with the inhibitory acetylation marks on Nrf2.

The identification of BET proteins as Nrf2 repressors adds another facet to an increasingly complex picture of Nrf2 signaling and biology. Over the last few years several other Keap1-independent pathways of Nrf2 regulation have been described based on experiments in mammalian cell culture: for example it was shown that PKC-δ mediated phosphorylation of Ser40 of mammalian Nrf2 promotes its stabilization and nuclear translocation [57–59]. The Src-family tyrosine kinase Fyn can phosphorylate Tyr568 of Nrf2 causing its nuclear export and degradation [60]. Glycogen synthase kinase 3 beta (GSK-3β) can phosphorylate Fyn and increase its nuclear accumulation and thereby promotes nuclear export of Nrf2 and inhibition of Nrf2 signaling [61]. In addition, GSK-3β can phosphorylate the serine residues at the β-TRCP-binding motif (DSGIS^338^) in the Neh6 domain of Nrf2 and promote Cullin 1 dependent proteasomal degradation by β-TRCP [62]. Future experiments will help to evaluate whether the Fs(1)h and CncC interaction cooperates with any of these more recently described mechanisms of Nrf2 regulation.

## Materials and Methods

### Plasmids and fly lines

All plasmids were generated by standard recombinant DNA and PCR methods. The pUAS-HA-attB plasmid was generated by cloning attB sequence amplified with PattB-F and PattB-R primers into pUAS-HA plasmid [8]. UAS-Fs(1)h-L plasmid was generated in two steps. First the Fs(1)h short isoform cDNA was amplified from LD26482 cDNA clone with PFs(1)hS-UAST-HA-F and PFs(1)hS-UAS-HA-R primers and was cloned into pUAS-HA-attB plasmid to generate pUAS-Fs(1)h-S-HA-attB plasmid. Then the cDNA for the C-terminal motif of Fs(1)h-L was amplified from total *Drosophila* cDNA with PUAST-Fs(1)h-L-int-F and PUAST-Fs(1)hL-int-R primers and was cloned into pUAS-Fs(1)h-S-HA-attB plasmid to generate pUAS-Fs(1)hL-HA-attB plasmid. The primer sequences are provided in Table A in S1 Text.

UAS-Fs(1)h-L transgenic fly strain was generated by Genetic Services Inc, MA using ΦC31 recombinase-mediated site-directed transformation [63]. EP-Fs(1)h fly line #10097 (Bloomington Stock Center) was used to over-express Fs(1)h whereas v51227 and v108662 fly lines (Vienna Stock Center) were used to knock down Fs(1)h.

### Calcium phosphate transfection

*Drosophila* S2 cells and HEK293 cells were transiently transfected with plasmid constructs using the calcium phosphate method [11].

### Chemical and drug treatments

To study the effect of JQ1 on reporter gene expression *in vivo*, 5-day-old flies that were mated for one day and then separated into males and females, were fed food supplemented with 0.25 mM JQ1 (APExBIO) for 48 hours. 15–20 flies were used in each group for these experiments and 3–5 representative flies were chosen randomly for imaging. To assess the effect of JQ1 on the cell-based reporters, S2 cells were transiently transfected with the reporter plasmids by the calcium phosphate method. 8 hours after the PBS wash and medium change, the transfected cells were transferred to 96-well plates and treated with 1 μM JQ1 and were incubated at 25°C for 24 hrs. Treatment with oltipraz was done following the same protocol [11]. In order to study the effect of different chemicals on the ARE reporter in mammalian cells, transfected 293T cells were treated with 0.5μM JQ1 and 10μM sulforaphane (Enzo Life Sciences) and were incubated at 37°C for 18hrs. DMSO was used as the solvent control in all the drug treatments.

### dsRNA synthesis and treatment

dsRNAs (200–700 bp) were synthesized and purified following the protocol provided as described [64] using ‘T7 RiboMAX Express RNAi System’ kit (Promega) and ‘RNeasy Kit’ (QIAGEN). Briefly, 1X10^6^ S2 cells were bathed with 8μg dsRNAs and these cells were transfected with luciferase reporter plasmids using the calcium phosphate method 3 days after dsRNA treatment. Predesigned dsRNAs obtained through the E-RNAi webservice [65] were used to knock down Fs(1)h, CncC, Keap1 whereas Fs(1)h-L was knocked down using the dsRNA described by Kockmann et al. [29]. dsRNA targeting the unique 3’ UTR region of Fs(1)h-S was designed by the ‘SnapDragon’ webservice. The sequences of primers used to generate amplicons for dsRNA synthesis are provided in Table B in S1 Text.

### Luciferase assay

The ‘Dual Glo Luciferase Assay System’ kit (Promega) was used to measure the activities of cell-based firefly and renilla luciferase reporters.

### Oxidative stress sensitivity assay

Oxidative stress resistance of adult flies of different genotypes was assessed as previously described [8]. Newly emerged flies of the specified genotypes were mated for one day, separated into females and males, and at 5 days of age were transferred to RU486-containing (300μM) or control food containing the solvent (ethanol). After 4 days 4 groups of 20 flies from each condition were starved for 3 hours in empty vials, and then fed a solution of 5% sucrose ± a semi-lethal dose of DEM (20mM). Survivors were scored after 36 hours and Log-rank tests were performed on the survivorship data using ‘GraphPad Prism’ software. In order to study the synergistic effect of oltipraz and JQ1 on stress sensitivity, flies raised on food supplemented with 0.4mM oltipraz and/or 0.1mM JQ1 for four days were exposed to similar DEM treatment and the survivorship data was collected and analyzed as before.

### Microscopy

To study the effect of Fs(1)h knock down and Fs(1)h-L over-expression on ARE activity in clones of tissue hsFlp; Act>Y>Gal4, UAS-RFP, ARE-GFP; UAS-Fs(1)h-RNAi and hsFlp; Act>Y>Gal4, UAS-RFP, ARE-GFP; UAS-Fs(1)h-L flies were used. The embryos and larvae were incubated at 18°C before L2 larvae were heat treated at 37°C for 30 minutes and then returned to 18°C. Adult flies were dissected in PBS and crops and ejaculatory bulbs were fixed at room temperature for 30 minutes in 100mM glutamic acid, 25mM KCl, 20mM MgSO_4_, 4mM sodium phosphate, 1mM MgCl_2_, and 4% formaldehyde (pH 7.5). DNA was stained with Hoechst dye. Confocal images were collected using a Leica TCS SP5 system and were processed using Adobe Photoshop.

### Co-immuno-precipitation and western blot

S2 cells transfected with pAct-Gal4 and pUAS-CncC-FLAG plasmids were harvested and washed with cold 1XPBS. The cells were then re-suspended in lysis buffer (50mM Tris-HCl, 200mM NaCl, 5mM EDTA, 5% Glycerol, 0.2% NP-40) that contained protease inhibitor complex (Roche) and kept on a nutator at 4°C for 1 hour. The cell debris was removed by centrifugation and the lysate was passed through hypodermic syringe to shear DNA. The protein concentration was estimated using Bradford’s reagent and 20–30 μg of protein from each sample was set aside as input. The lysate was pre-cleared with Protein G beads (GE Biosciences) for 1 hour at 4°C. The beads were separated by centrifugation and the cleared lysate was transferred into the fresh chilled tube. Anti-FLAG (Sigma-Aldrich Co) antibody was added at 2 μg per mg lysate protein concentration and was incubated at 4°C for 2 hours. Then 20 μl of 50% Protein G bead slurry was added to the lysate and incubated overnight on rotating disc at 4°C. The beads were spun down and were washed 5 times with 750 ul of cold lysis buffer. After the final wash, 7.5 μl of sample buffer and 7.5 μl of lysis buffer were added to the beads and incubated at 95°C along with the input samples for 10 minutes. The beads were spun down at the top speed for 5 minutes and the supernatant was loaded for electrophoresis. For LBH and JQ1 treatment, the cells were treated with 2μM of LBH (APExBIO) and 10 μM JQ1 for 6 hours prior to the cell lysis. JQ1 was also added to the lysate from cells treated with JQ1 at 10 μM concentration after the cell lysis. Primary antibody specific to Fs(1)h-L isoform (1:2000 dilution) (Kindly provided by Dr. Victor Corces) [28] and secondary anti-rabbit antibody (1:5000 dilution) (BioRad) were used to probe the western blot membrane to examine the binding of Fs(1)h-L protein to CncC-FLAG protein. Antibody that recognizes both isoforms of Fs(1)h (Kindly provided by Dr. Igor Dawid) was used in 1:2000 dilution in western blot experiments to validate the selective knock down of Fs(1)h-S and Fs(1)h-L with isoform-specific dsRNAs. Antibody against acetylated lysine (Cell Signaling Technology) was used in (1:1000) concentration.

### Quantitative RT-PCR

mRNA was prepared from whole flies or from S2 cells using Trizol reagent (Invitrogen). DNaseI (NEB) was used to remove contaminating DNA before phenol: chloroform: isoamyl alcohol (25:24:1, Amresco) extraction. Maxima reverse transcriptase (Fermentas) and oligo dT primers were used to generate cDNA. cDNA was diluted 1:50 to serve as template for quantitative real time PCR (qPCR). qPCR reactions were done in triplicates using qPCR super-mix (BioRad) on a BioRad MyIQ thermal cycler. ‘Delta-delta Ct’ method was used for normalization to *actin5c* transcript levels. Data shown are averages and standard deviations from at least three biological replicates. The sequences of primers used to qPCR are provided in Table C in S1 Text.

## Supporting Information

S1 Fig Validation of knock down and over-expression conditions of Fs(1)h. (A) Both Fs(1)h isoforms can be effectively and selectively knocked down in S2 cells. Treatment of S2 cells with dsRNA targeting the C-terminal motif of the long isoform (Fs(1)h-L^RNAi^) selectively depleted Fs(1)h-L whereas dsRNA targeting the 3’-UTR of Fs(1)h-S (Fs(1)h^RNAi^) led to selective depletion of Fs(1)h-S. dsRNA targeting gene region shared by both isoforms (Fs(1)h^RNAi^) knocked down both isoforms of Fs(1)h. (B) Validation of Fs(1)h over-expression in P(EP)*fs(1)h*[EP439] and Fs(1)h knock down in UAS-Fs(1)h^RNAi^ (v51227). qPCR experiments with primers that can detect both isoforms of Fs(1)h showed that RU486 treatment causes an increase of Fs(1)h mRNA in tub-GS-Gal4>EP-Fs(1)h flies and a knock down of Fs(1)h mRNA in tub-GS-Gal4>Fs(1)h^RNAi^ flies. The mRNA levels were normalized to *actin5C* transcript levels. The error bars indicate standard deviation of 3 biological replicates.
(TIF)

S2 Fig *fs(1)h* gain- and loss-of-function conditions affect ARE reporter activity in *Drosophila*. (A) Ubiquitous expression of Fs(1)h-L from a UAS-driven transgene in adult *Drosophila* suppresses ARE reporter activity. RU486-induced over-expression of Fs(1)h-L in tub-GS-Gal4, ARE-GFP; UAS-Fs(1)h-L flies reduced oltipraz-induced ARE-GFP reporter activity in the whole body. (B) Ubiquitous knock down of Fs(1)h using an additional RNAi line (v108662) that targets a different part of the Fs(1)h gene than the one shown in Fig 2A, using the RU486-inducible tub-GS-Gal4 driver stimulates ARE-GFP reporter activity in most tissues of adult flies. (C) Ubiquitous expression of Fs(1)h-L from a UAS-driven transgene in adult *Drosophila* suppresses ARE reporter activity. RU486 induced over-expression of Fs(1)h-L in tub-GS-Gal4,ARE-GFP;UAS-Fs(1)h-L flies reduced oltipraz induced gstD-GFP reporter [8] activity in the whole body. Two RU486-treated and two mock-treated females that were randomly chosen are shown in all panels.
(TIF)

S3 Fig *fs(1)h* gain- and loss-of-function conditions affect oxidative stress resistance in adult flies. (A) Fs(1)h-L was over-expressed in tub-GS-Gal4; UAS-Fs(1)h-L male and female adults by maintaining them on food containing 300μM RU486 for 4 days. Lethality after exposure to 20μM DEM was recorded and analyzed by Mantel-Cox log-rank test. The flies on RU486 food, showed significantly increased sensitivity to DEM (P value < 0.005 for females and P value < 0.05 for males) compared to those on control food. (B) Fs(1)h was over-expressed in male EP-fs(1)h; tub-GS flies by exposing them to food containing 300μM RU486 for 4 days. Lethality after exposure to 20μM DEM was recorded and analyzed by Mantel-Cox log-rank test. The flies on RU486 food, showed significantly increased sensitivity to DEM (P value < 0.05) compared to those on control food. (C) Ubiquitous Fs(1)h knock-down in young adult males by inducible expression of UAS-Fs(1)h^RNAi^ transgene under the control of the tub-GS-Gal4 driver. Survival after exposure to 20μM DEM was recorded and the data were analyzed by Mantel-Cox log-rank test. Male flies reared on RU486 food, showed significantly increased resistance to DEM (P value <0.001) compared to those on control food. (D) *w*^*1118*^ male flies were maintained on food containing 0.4mM oltipraz and/or 0.1mM JQ1 for 4 days and then were exposed to 20μM DEM. Survivorship was assessed. Mantel-Cox log-rank test showed that combinatorial pre-treatment with oltipraz and JQ1 extended survival after DEM exposure significantly more than pre-treatment with either drug alone (P value <0.001 for oltipraz/combined comparison and P value <0.005 for JQ1/combined comparison). Oxidative stress tolerance was also significantly enhanced by pre-treatment with either oltipraz or JQ1 (P value <0.005 for both control/oltipraz and control/JQ1 comparisons).
(TIF)

S4 Fig Fs(1)h-L specifically interacts with the C isoform of Cnc. Co-immuneprecipitation (IP) experiment of Fs(1)h and different Cnc isoforms. *Drosophila* S2 were cells transfected with plasmids expressing Flag-tagged CncA, CncB, or CncC, as indicated. Cells were lysed 36 h after transfection; 90% of the lysates were used for IP with anti-Flag antibody and 10% of the lysates were used as input. The cell lysates and immuno-precipitates were analyzed by immuno-blotting (IB) with anti-Fs(1)h or anti-Flag antibodies as indicated. Arrows indicate the position of bands corresponding to Flag-CncA, B, C, Fs(1)h-S and Fs(1)h-L, respectively.
(TIF)

S5 Fig BET proteins regulate Nrf2 activity independently of Keap1. (A) Combination of Fs(1)h knock down with either Keap1 knock down or CncC over-expression under Actin-Gal4 driver causes synergistic activation of ARE-Fluc reporter in S2 cells. Over-expression of Keap1, on the other hand, suppresses induction of ARE in response to Fs(1)h knock down. (B) Combination of BET protein inhibitor JQ1(0.5μM) but not Keap1 inhibitor sulforaphane (10μM) with transient Nrf2 over-expression from a CMV-driven expression vector causes synergistic activation of NQO1-fluc reporter in HEK293 cells.
(TIF)

S1 Document Variability in oxidative stress responsiveness among biological replicates. The stress sensitivity experiments were carried out with 4 biological replicates (separate vials, each with 20 flies). The error bars represent standard deviations in percent survival among biological replicates.
(PDF)

S1 Text Primer sequences used in cloning, dsRNA synthesis and qPCR. (DOCX)
