# Supplementary figures and images for: Keap1-Independent Regulation of Nrf2 Activity by Protein Acetylation and a BET Bromodomain Protein
Source: PLoS Genet. 2016 May 27;12(5):e1006072. doi: 10.1371/journal.pgen.1006072 (PMC4883770; doi:10.1371/journal.pgen.1006072)

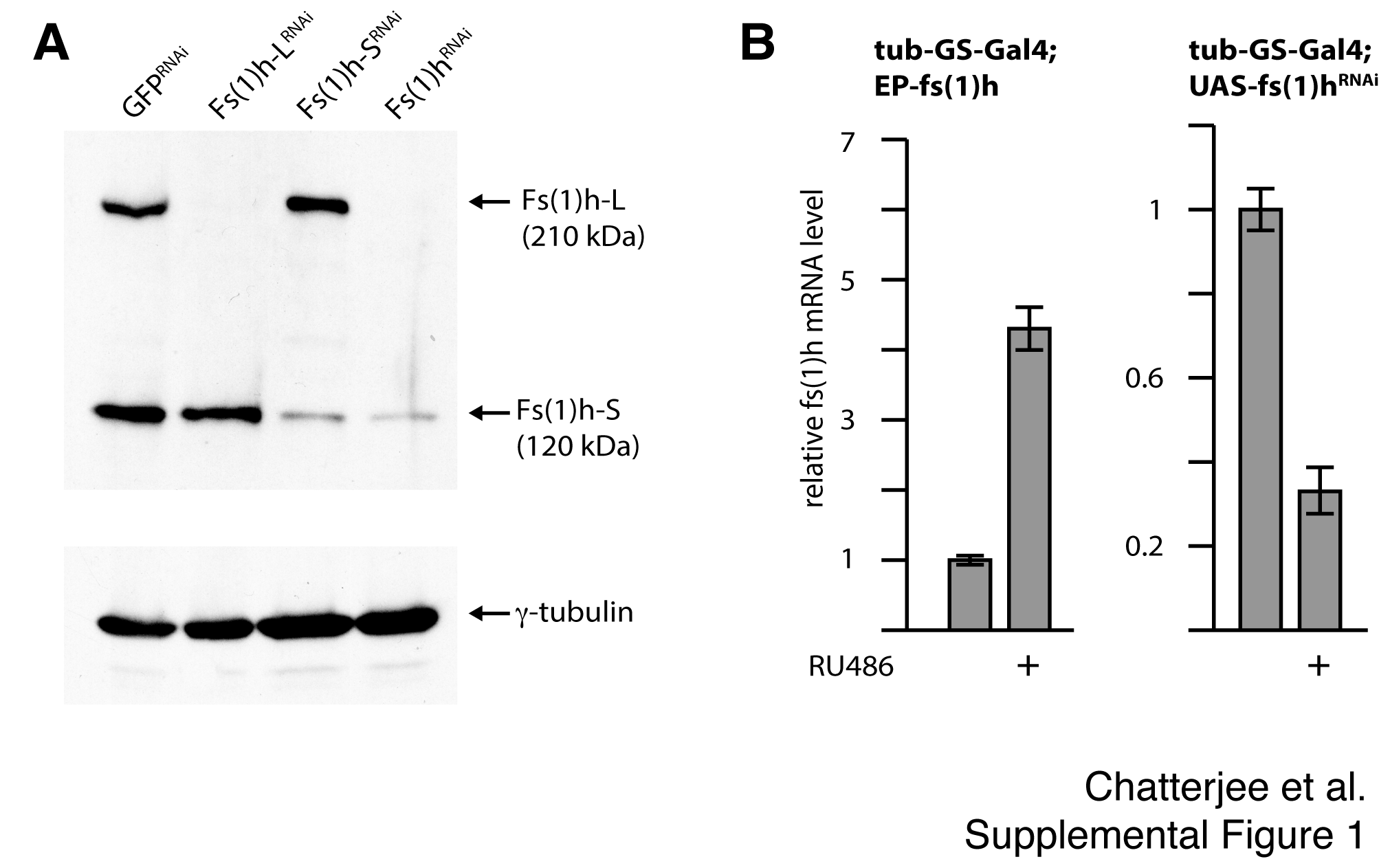

Supplement: S1 Fig — (A) Both Fs(1)h isoforms can be effectively and selectively knocked down in S2 cells. Treatment of S2 cells with dsRNA targeting the C-terminal motif of the long isoform (Fs(1)h-LRNAi) selectively depleted Fs(1)h-L whereas dsRNA targeting the 3’-UTR of Fs(1)h-S (Fs(1)hRNAi) led to selective depletion of Fs(1)h-S. dsRNA targeting gene region shared by both isoforms (Fs(1)hRNAi) knocked down both isoforms of Fs(1)h. (B) Validation of Fs(1)h over-expression in P(EP)fs(1)h[EP439] and Fs(1)h knock down in UAS-Fs(1)hRNAi (v51227). qPCR experiments with primers that can detect both isoforms of Fs(1)h showed that RU486 treatment causes an increase of Fs(1)h mRNA in tub-GS-Gal4>EP-Fs(1)h flies and a knock down of Fs(1)h mRNA in tub-GS-Gal4>Fs(1)hRNAi flies. The mRNA levels were normalized to actin5C transcript levels. The error bars indicate standard deviation of 3 biological replicates. (TIF) [file pgen.1006072.s001.tif]

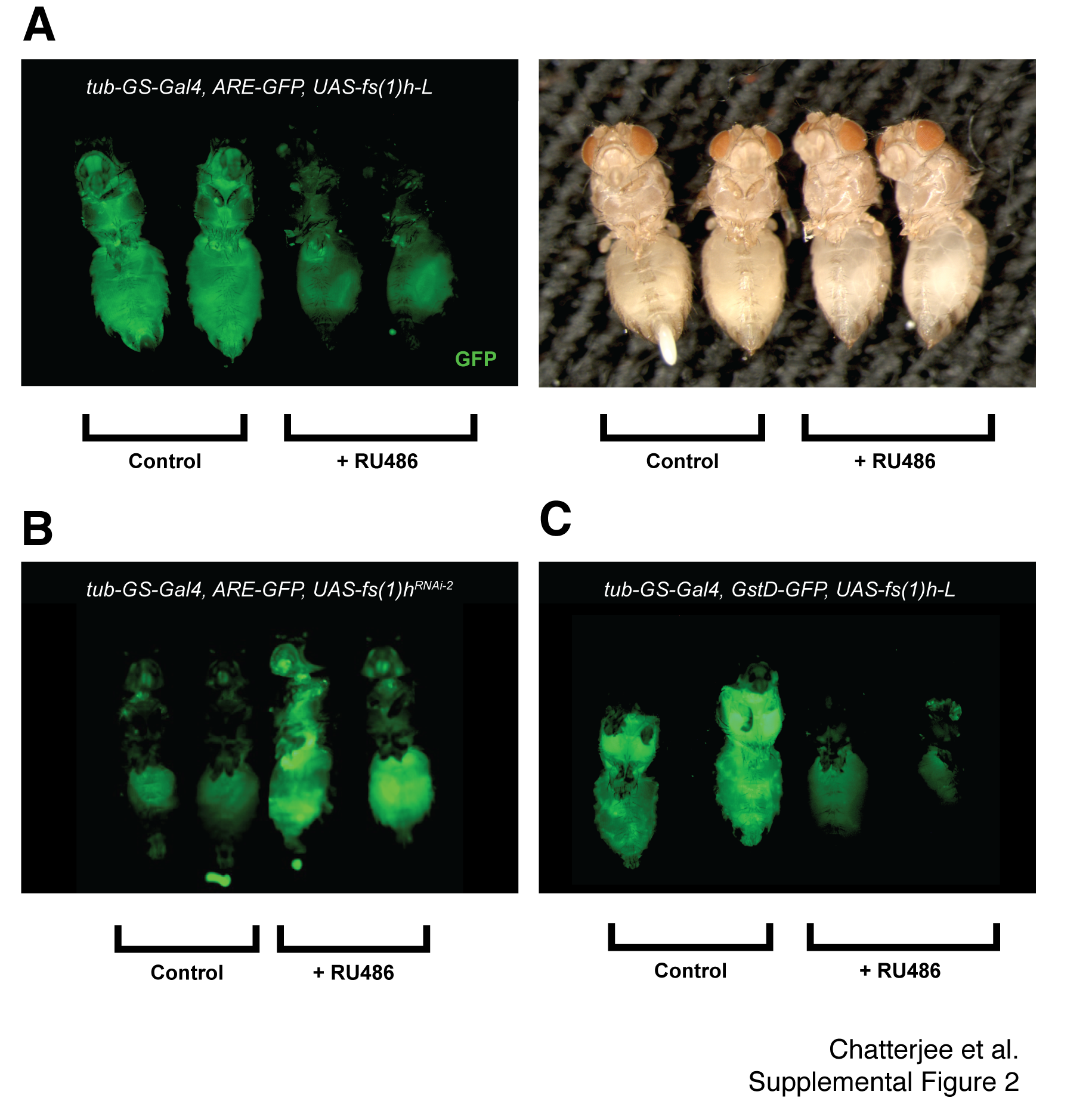

Supplement: S2 Fig — (A) Ubiquitous expression of Fs(1)h-L from a UAS-driven transgene in adult Drosophila suppresses ARE reporter activity. RU486-induced over-expression of Fs(1)h-L in tub-GS-Gal4, ARE-GFP; UAS-Fs(1)h-L flies reduced oltipraz-induced ARE-GFP reporter activity in the whole body. (B) Ubiquitous knock down of Fs(1)h using an additional RNAi line (v108662) that targets a different part of the Fs(1)h gene than the one shown in Fig 2A, using the RU486-inducible tub-GS-Gal4 driver stimulates ARE-GFP reporter activity in most tissues of adult flies. (C) Ubiquitous expression of Fs(1)h-L from a UAS-driven transgene in adult Drosophila suppresses ARE reporter activity. RU486 induced over-expression of Fs(1)h-L in tub-GS-Gal4,ARE-GFP;UAS-Fs(1)h-L flies reduced oltipraz induced gstD-GFP reporter [8] activity in the whole body. Two RU486-treated and two mock-treated females that were randomly chosen are shown in all panels. (TIF) [file pgen.1006072.s002.tif]

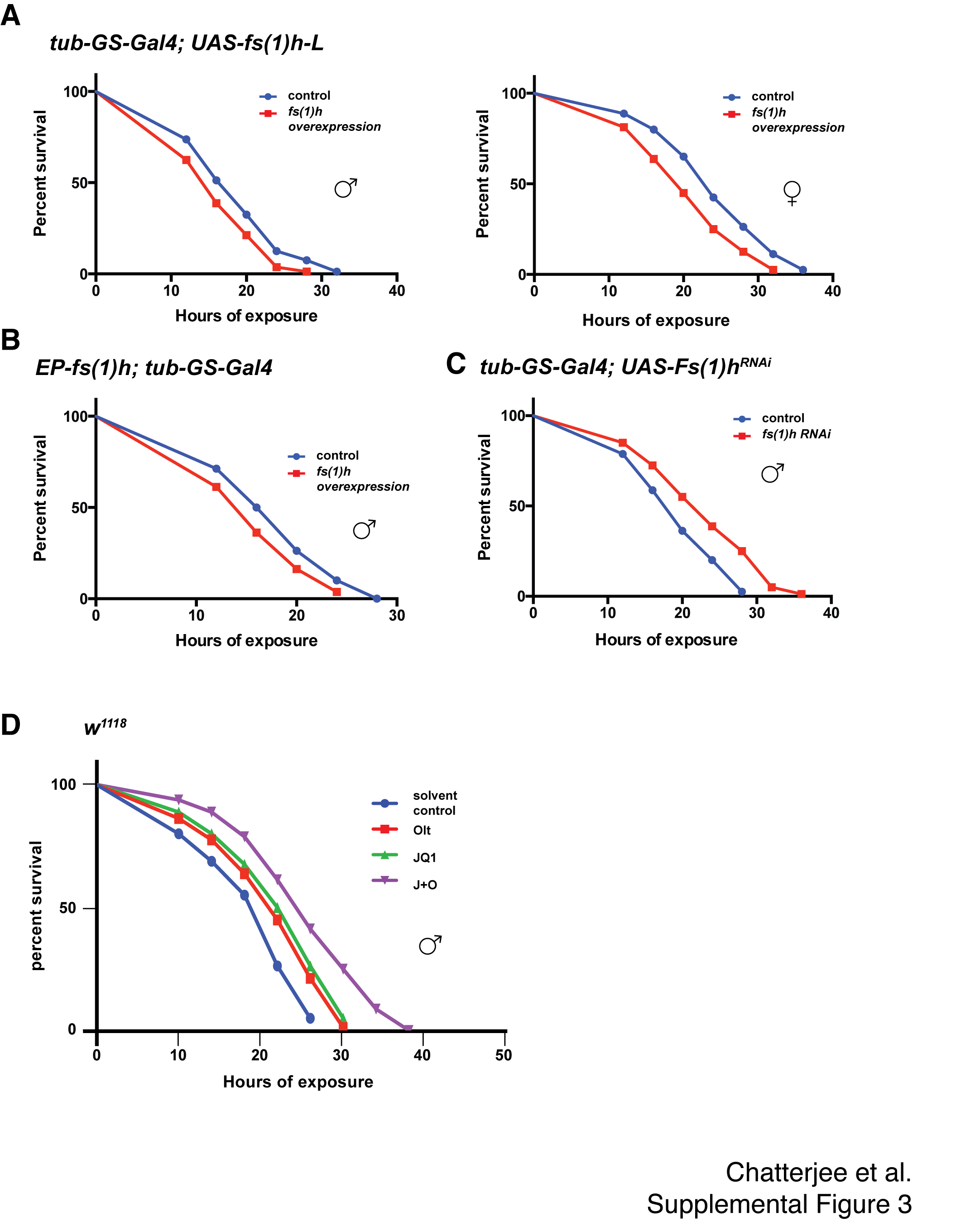

Supplement: S3 Fig — (A) Fs(1)h-L was over-expressed in tub-GS-Gal4; UAS-Fs(1)h-L male and female adults by maintaining them on food containing 300μM RU486 for 4 days. Lethality after exposure to 20μM DEM was recorded and analyzed by Mantel-Cox log-rank test. The flies on RU486 food, showed significantly increased sensitivity to DEM (P value < 0.005 for females and P value < 0.05 for males) compared to those on control food. (B) Fs(1)h was over-expressed in male EP-fs(1)h; tub-GS flies by exposing them to food containing 300μM RU486 for 4 days. Lethality after exposure to 20μM DEM was recorded and analyzed by Mantel-Cox log-rank test. The flies on RU486 food, showed significantly increased sensitivity to DEM (P value < 0.05) compared to those on control food. (C) Ubiquitous Fs(1)h knock-down in young adult males by inducible expression of UAS-Fs(1)hRNAi transgene under the control of the tub-GS-Gal4 driver. Survival after exposure to 20μM DEM was recorded and the data were analyzed by Mantel-Cox log-rank test. Male flies reared on RU486 food, showed significantly increased resistance to DEM (P value <0.001) compared to those on control food. (D) w1118 male flies were maintained on food containing 0.4mM oltipraz and/or 0.1mM JQ1 for 4 days and then were exposed to 20μM DEM. Survivorship was assessed. Mantel-Cox log-rank test showed that combinatorial pre-treatment with oltipraz and JQ1 extended survival after DEM exposure significantly more than pre-treatment with either drug alone (P value <0.001 for oltipraz/combined comparison and P value <0.005 for JQ1/combined comparison). Oxidative stress tolerance was also significantly enhanced by pre-treatment with either oltipraz or JQ1 (P value <0.005 for both control/oltipraz and control/JQ1 comparisons). (TIF) [file pgen.1006072.s003.tif]

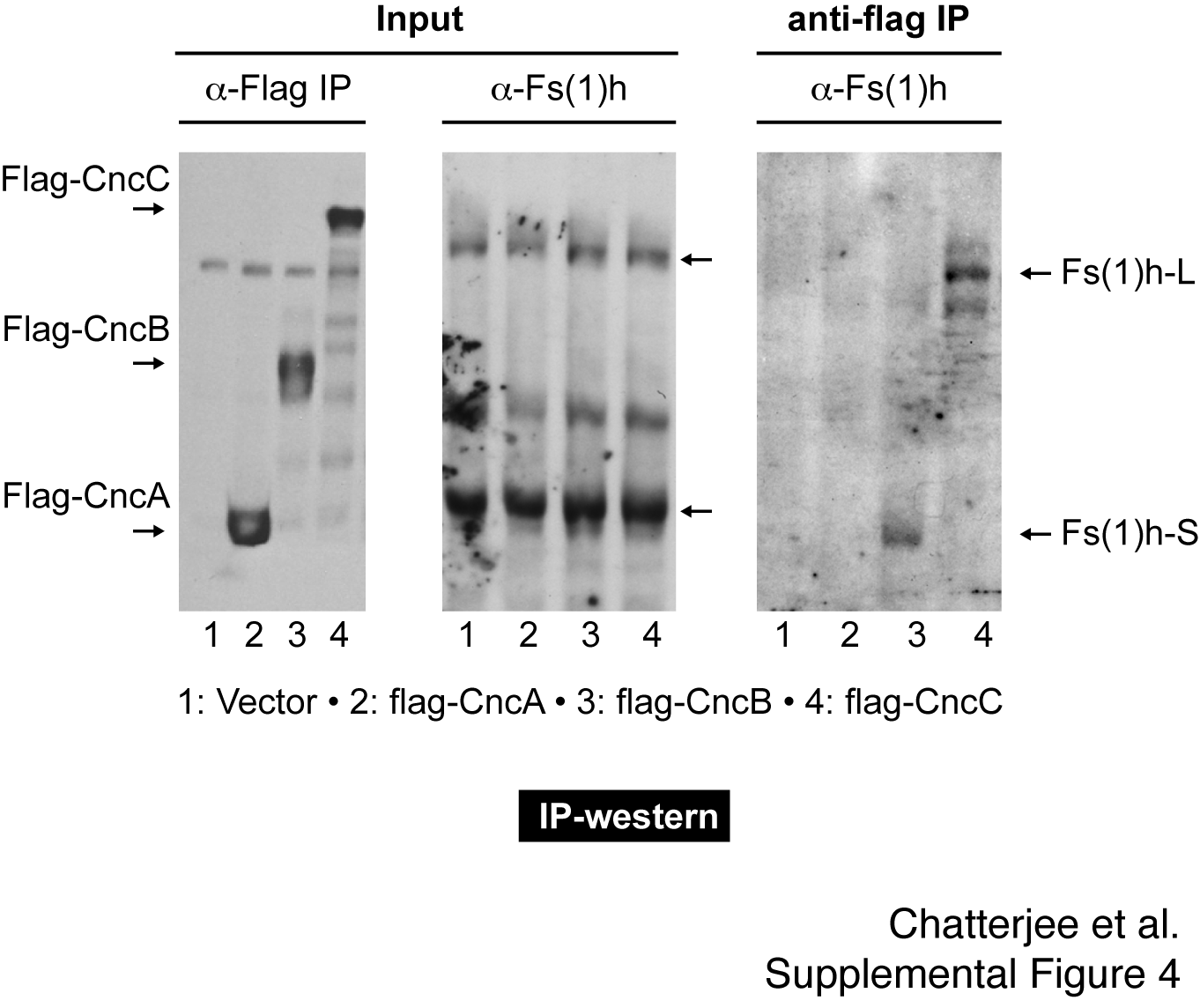

Supplement: S4 Fig — Co-immuneprecipitation (IP) experiment of Fs(1)h and different Cnc isoforms. Drosophila S2 were cells transfected with plasmids expressing Flag-tagged CncA, CncB, or CncC, as indicated. Cells were lysed 36 h after transfection; 90% of the lysates were used for IP with anti-Flag antibody and 10% of the lysates were used as input. The cell lysates and immuno-precipitates were analyzed by immuno-blotting (IB) with anti-Fs(1)h or anti-Flag antibodies as indicated. Arrows indicate the position of bands corresponding to Flag-CncA, B, C, Fs(1)h-S and Fs(1)h-L, respectively. (TIF) [file pgen.1006072.s004.tif]

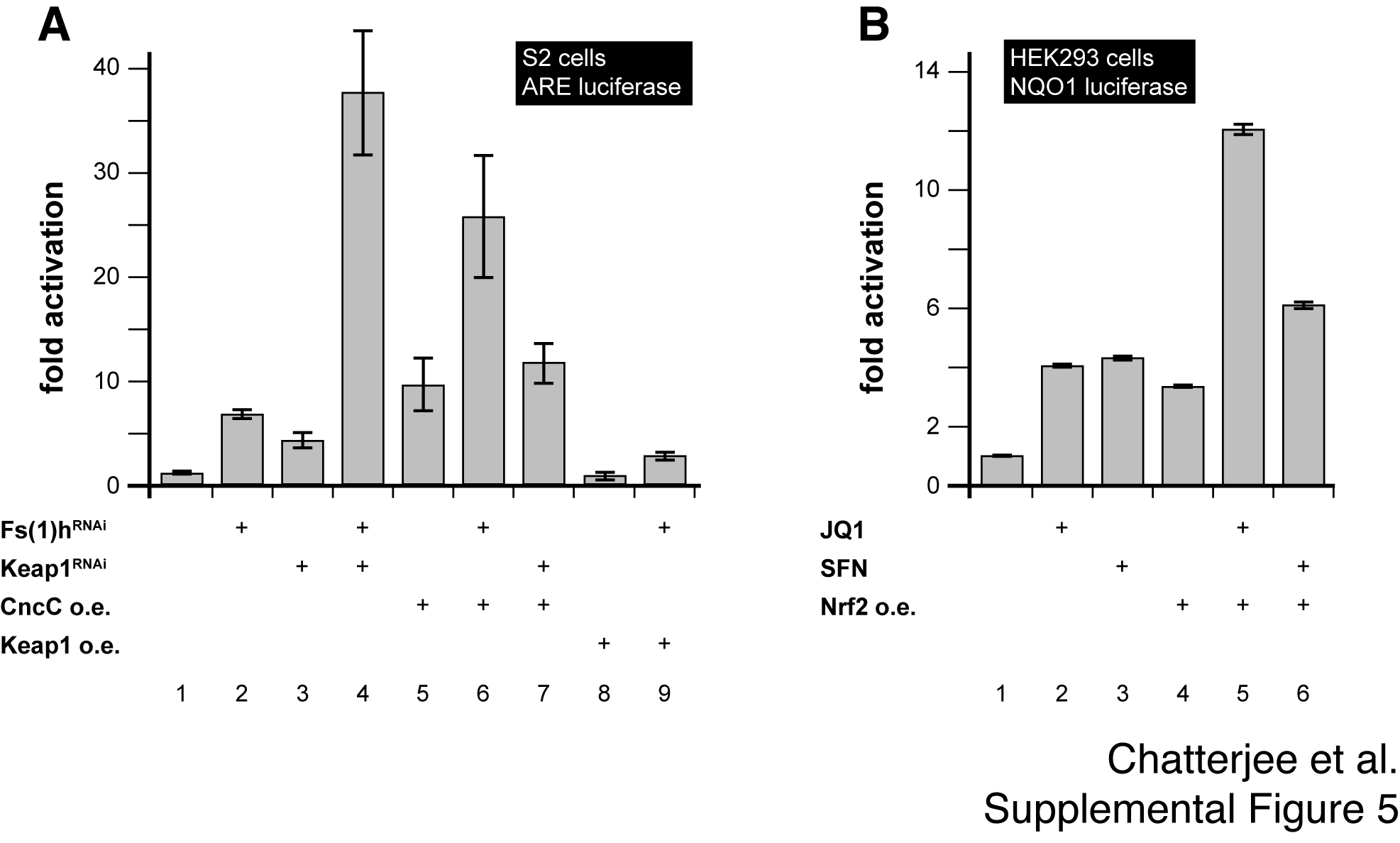

Supplement: S5 Fig — (A) Combination of Fs(1)h knock down with either Keap1 knock down or CncC over-expression under Actin-Gal4 driver causes synergistic activation of ARE-Fluc reporter in S2 cells. Over-expression of Keap1, on the other hand, suppresses induction of ARE in response to Fs(1)h knock down. (B) Combination of BET protein inhibitor JQ1(0.5μM) but not Keap1 inhibitor sulforaphane (10μM) with transient Nrf2 over-expression from a CMV-driven expression vector causes synergistic activation of NQO1-fluc reporter in HEK293 cells. (TIF) [file pgen.1006072.s005.tif]
