# Supplementary material for: Keap1-Independent Regulation of Nrf2 Activity by Protein Acetylation and a BET Bromodomain Protein
Source: PLoS Genet. 2016 May 27;12(5):e1006072. doi: 10.1371/journal.pgen.1006072 (PMC4883770; doi:10.1371/journal.pgen.1006072)
Supplement: S1 Document — The stress sensitivity experiments were carried out with 4 biological replicates (separate vials, each with 20 flies). The error bars represent standard deviations in percent survival among biological replicates. (PDF) [file pgen.1006072.s006.pdf]

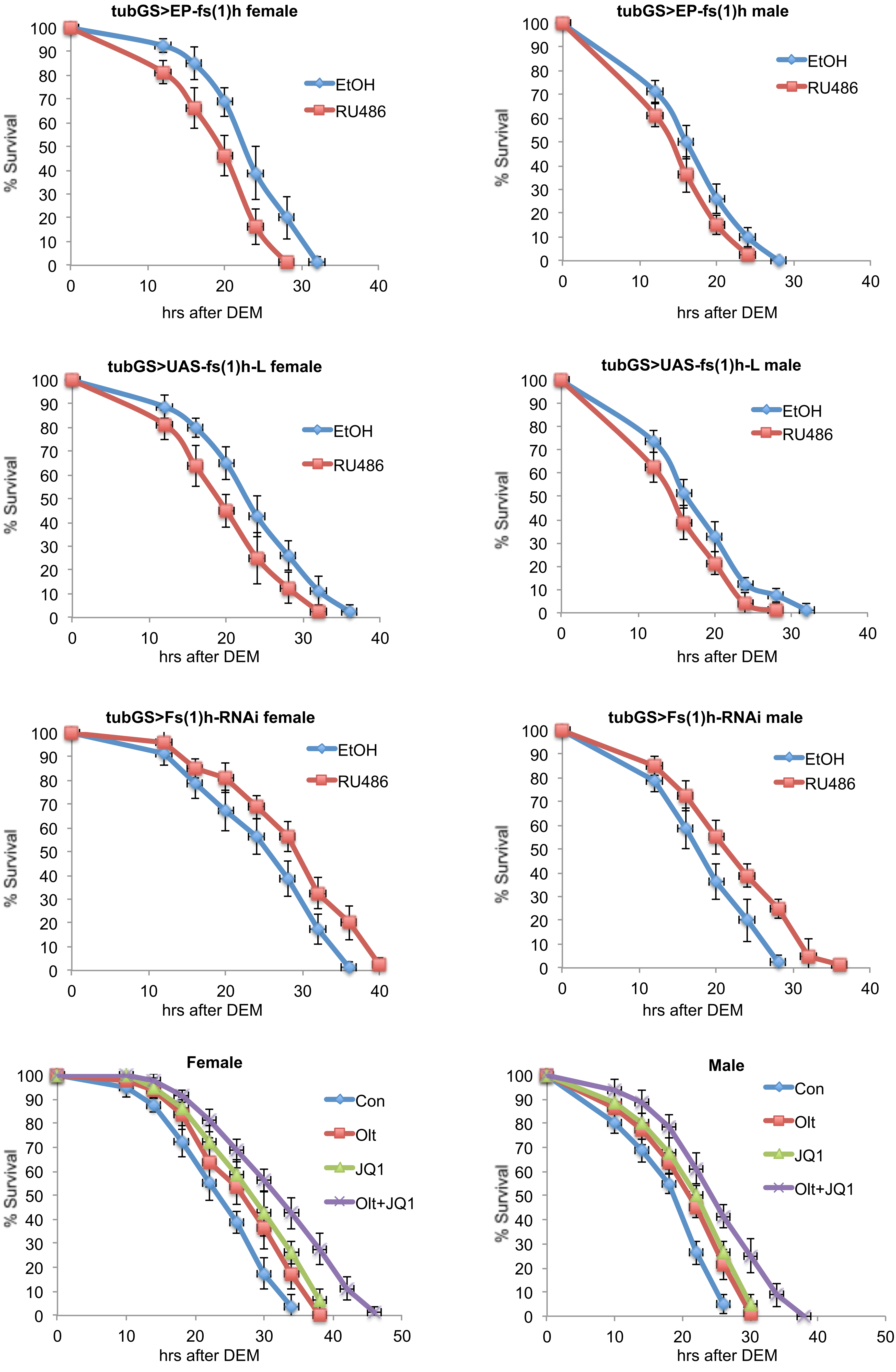

Chatterjee et al.
Supplemental Document
