# Supplementary material for: Keap1-Independent Regulation of Nrf2 Activity by Protein Acetylation and a BET Bromodomain Protein
Source: PLoS Genet. 2016 May 27;12(5):e1006072. doi: 10.1371/journal.pgen.1006072 (PMC4883770; doi:10.1371/journal.pgen.1006072)
Supplement: S1 Text — (DOCX) [file pgen.1006072.s007.docx]

**Table A: Primers used in pUAS-Fs(1)hL-HA-attB cloning**

| **Primer Name** | **Primer sequence** |
| --- | --- |
| PattB-F | GGAATTCCATATGATTACGCCAAGCGCGCA |
| PattB-R | GGAATTCCATATGCAGCCCAAGCTTATCGATAC |
| PFs(1)h-S-UAST-HA-F | CGGAATTCTGAAGCACCAGAACCACCAC |
| PFs(1)h-S-UAST-HA-R | GAAGATCTATACCTGCTTCACTGTCGCTCGA |
| PUAST-Fs(1)h-L-int-F | TGGAGAAGCGCCTGCAGGACGTC |
| PUAST-Fs(1)h-L-int-R | GAAGATCTATCAGCGTCTCCTCAAAGGC |

**Table B: Primers for dsRNA synthesis**

| **Primer name** | **Primer sequence** |
| --- | --- |
| Fs(1)hi-F | TTCCTCATCCGAGTTGGATT |
| Fs(1)hi-R | TGAACAAGGAGAAGCTGTCGG |
| Fs(1)h-Si-F | GCGTGGCTTGAGTCAGAGA |
| Fs(1)h-Si-R | CTCTTGGGCGTGCTAATGGT |
| Fs(1)h-Li-F | GGGCAGCAGCACAACAAGAAT |
| Fs(1)h-Li-R | GGTAGAACCGGCGCTGAACT |
| Keap1i-F | CGTGTTGTGGTACTCCATGC |
| Keap1i-R | CCAACTTCCTCAAGGAGCAG |
| CncCi-F | TCAATGTGCTACCTTATTGACT |
| CncCi-R | TTATCTTGTTGAAGCTCCTCC |
| MafSi-F | GATTTGGTGAGCATTTCGGT |
| MafSi-R | AAACAGGAATGTGACTGGGC |
| GFPi-F | CACATGAAGCAGCACGACTT |
| GFPi-R | TGTTCTGCTGGTAGTGGTCG |

Each dsRNA primer sequence is preceded by a T7 promoter sequence (TAATACGACTCACTATAGG) at the 5’ end.

**Table C: Primers used in qPCR experiments**

| **Primer name** | **Primer sequence** |
| --- | --- |
| Fs(1)h-FP | AACAAGTTGCCAGGGGACAA |
| Fs(1)h-RP | GATTGGAGTCACGCAGCGAT |
| GclC-FP | CGCTTTATCCAGAAGCGTGCCG |
| GclC-RP | AATCCTGCTTGTAATCCGGGTGGC |
| GstD1-FP | GGCCGCCTTCGAGTTCCTGA |
| GstD1-RP | CGGTTGCCACCAGGGCAATG |
| GstE9-FP | GGTGCCGCGTTCCCAGATTG |
| GstE9-RP | GACCGGTCCGCAGAGGTAAGC |
| Keap1-FP | TGGCCAGCGTGGAGTGCTAC |
| Keap1-RP | TTGCAGCAACACCCGCTCCA |
| Act5C-FP | CCTGGCATCGCCGACCGTAT |
| Act5C-RP | AGTACTTGCGCTCTGGCGGG |
